# Do large language models have a legal duty to tell the truth?

**DOI:** 10.1098/rsos.240197

**Published:** 2024-08-07

**Authors:** Sandra Wachter, Brent Mittelstadt, Chris Russell

**Affiliations:** Oxford Internet Institute, University of Oxford, 1 St Giles, Oxford OX1 3JS, UK

**Keywords:** truth, artificial intelligence, human rights, law, philosophy, large language models

## Abstract

Careless speech is a new type of harm created by large language models (LLM) that poses cumulative, long-term risks to science, education and shared social truth in democratic societies. LLMs produce responses that are plausible, helpful and confident, but that contain factual inaccuracies, misleading references and biased information. These subtle mistruths are poised to cumulatively degrade and homogenize knowledge over time. This article examines the existence and feasibility of a legal duty for LLM providers to create models that ‘tell the truth’. We argue that LLM providers should be required to mitigate careless speech and better align their models with truth through open, democratic processes. We define careless speech against ‘ground truth’ in LLMs and related risks including hallucinations, misinformation and disinformation. We assess the existence of truth-related obligations in EU human rights law and the Artificial Intelligence Act, Digital Services Act, Product Liability Directive and Artificial Intelligence Liability Directive. Current frameworks contain limited, sector-specific truth duties. Drawing on duties in science and academia, education, archives and libraries, and a German case in which Google was held liable for defamation caused by autocomplete, we propose a pathway to create a legal truth duty for providers of narrow- and general-purpose LLMs.

## 1. Introduction

Large language models (LLM) and other generative AI systems pose new risks and opportunities for society. Risks such as bias, environmental impact, privacy issues, misinformation and the problem of hallucinations stem from how these models are built and operate, but others arise from our relationship with the technology. While problems arising from our tendency to anthropomorphize machines are well established [1,2], our vulnerability to treating LLMs as human-like truth tellers is uniquely worrying.

Popular LLMs such as ChatGPT and Gemini are text-generation engines designed to predict which string of words comes next in a piece of text. They are fine-tuned via ‘reinforcement learning from human feedback’ (RLHF) to make their outputs more human-like, persuasive and useful to users that ask them questions or provide prompts requesting generation of text, images, code, video or other media [3,4].

LLMs are not designed to tell the truth in any overriding sense. They frequently stray far from the truth or ‘hallucinate’ in their quest to be convincing and helpful to users, but equally are prone to produce small mistruths, oversimplifications of complex topics and responses biased towards certain commonly held beliefs or schools of thought. These are, strictly speaking, an effect of design choices taken by LLM providers. Truthfulness or factuality is only one performance measure among many others such as ‘helpfulness, harmlessness, technical efficiency, profitability, [and] customer adoption’ [5,6]. RLHF similarly introduces latent performance measures and biases derived from the annotator feedback and ranking, such as a preference for assertive sounding outputs [7], or content that aligns with prior beliefs (referred to as ‘sycophancy’) [8].

General-purpose LLMs will readily answer questions and produce outputs on any topics except for those which contravene human-designed ‘guardrails' that help to avoid sensitive or toxic content [9,10].^1^ Despite their generality, responses rarely include linguistic signals or measures of confidence [12,13]. Links to source material are provided by some systems such as Bing's integration of GPT-4, but are often incorrectly interpreted or referenced inappropriately [14,15]. Unlike human speakers, LLMs do not have any internal conceptions of expertise or confidence, instead always ‘doing their best’ to be helpful and persuasively respond to the prompt posed [16]. They are designed to participate in natural language conversations with people and offer answers that are convincing and feel helpful, regardless of the truth of the matter at hand ([5, p. 8], [16]).

When understood as a type of artificial speaker producing human-like language, the human tendency to attribute meaning and intent to natural language puts LLMs in a position to rapidly spread homogenized, oversimplified and non-representative knowledge at scale. The production of language implies that the speaker has understanding, intent, consciousness and ultimately intelligence [2,17–19]. This tendency leads users to focus more on the times LLMs ‘get it right’ and ignore hallucinations and subtle mistruths [16,20].

These effects are exacerbated by the habit of LLM providers and media to emphasize their power, using words that communicate human-like intelligence such as ‘knowledge’, ‘understanding’ or ‘self-learning’, [2,21] while at the same time warning about the eventual development of sentience, general human-like intelligence and ‘existential risks’.^2^ Users are both encouraged and innately susceptible to believing LLMs are telling the truth,^3^ but only meekly warned via easily missed notices and disclaimers that these systems are ‘experimental’ and that their outputs should not, in fact, be trusted as truthful (see table 1), such as a preference for assertive sounding outputs, or content that aligns with prior beliefs (referred to as ‘sycophancy’) [7,8].

**Table 1.** LLM notices and disclaimers.

LLM	description	disclaimer
ChatGPT	‘ChatGPT is an AI-powered language model developed by OpenAI, capable of generating human-like text based on context and past conversations’^114^	‘ChatGPT may produce inaccurate information about people, places or facts’^115^
Bard	‘Meet Bard: your creative and helpful collaborator, here to supercharge your imagination, boost your productivity, and bring your ideas to life’.^116^	‘Bard is an experiment and may give inaccurate or inappropriate responses. You can help make Bard better by leaving feedback’^117^
Gemini (formerly Bard)	‘Gemini gives you direct access to Google AI. Get help with writing, planning, learning, and more… When you enter a prompt into Gemini, it replies with a response using the information it already knows or fetches from other sources, like other Google services’	Landing page: ‘Gemini may display inaccurate info, including about people, so double-check its responses’.^118^ FAQ: ‘Gemini can hallucinate and present inaccurate information as factual…Gemini will make mistakes. Even though it's getting better every day, Gemini can provide inaccurate information, or it can even make offensive statements…Gemini has tools to help you identify potentially inaccurate statements. One way to double-check Gemini's responses is to use the Google button. This uses Google Search to find content that helps you assess and further research the information you get from Gemini…Gemini's double-check feature can make mistakes. For example, the feature may show that Google Search found content that makes a similar statement to Gemini's. But the content may actually contradict Gemini. The web content may be inaccurate, too. You should read, review, and carefully evaluate the content identified by the double-check feature, as well as its context’^119^
Bing (Copilot)	‘The new Bing is like having a research assistant, personal planner, and creative partner at your side whenever you search the web. With this set of AI-powered features, you can: Ask your actual question. When you ask complex questions, Bing gives you detailed replies. Get an actual answer. Bing looks at search results across the web to offer you a summarized answer’^120^	‘Bing aims to base all its responses on reliable sources—but AI can make mistakes, and third party content on the Internet may not always be accurate or reliable. Bing will sometimes misrepresent the information it finds, and you may see responses that sound convincing but are incomplete, inaccurate or inappropriate. Use your own judgement and double check the facts before making decisions or taking actions based on Bing's responses’^121^
LLaMa 2	LLaMa 2 is not a consumer facing chatbot, but a collection of LLMs that can easily be used to create a chatbot by AI developers. According to Meta: ‘Llama 2 was pretrained on publicly available online data sources. The fine-tuned model, Llama-2-chat, leverages publicly available instruction datasets and over 1 million human annotations’^122^	no disclaimer, but AI developers are directed to a Responsible Use Guide and an Acceptable Use Policy which requires developers to ensure LLaMa is not used to ‘Intentionally deceive or mislead others, including use of Llama 2 related to the following: a. Generating, promoting or furthering fraud or the creation or promotion of disinformation…Representing that the use of Llama 2 or outputs are human-generated’^123^

When paired with automation bias and technology bias, or the human tendency to attribute superior capabilities to technology [27], these trends point towards a new type of epistemic harm that emerges through the proliferation of trusted but epistemologically flawed machine-generated content in human discourse, beliefs, culture and knowledge. Identifying when this harm arises, how severe it is, why it occurs, what its long-term effects are on individual users and society, and how to fix them is extremely difficult [16,28].

This article takes a first step down this path to mitigate the homogenization and oversimplification of knowledge driven by LLMs. Obvious hallucinations are not the primary epistemic risk created by LLMs. As we have argued elsewhere, ‘it is subtle inaccuracies, oversimplifications or biased responses [29] that are passed off as truth in a confident tone—which can convince experts and non-experts alike—that pose the greatest risk’ [16,30,31]. Drawing on philosophy of science, Frankfurt's concept of ‘bullshit’ [32] and recent scholarship on post-truth politics [33,34], we conceptualize this type of problematic output from LLMs as ‘careless speech’. Unlike related concepts of misinformation, disinformation, libel and hallucinations in LLMs, careless speech causes unique long-term harms to science, education and society which resist easy quantification, measurement and mitigation. Voluntary technical measures to better align LLMs with ‘ground truth’ or reliable sources can help to combat these harms but are insufficient on their own. To address this gap and better mitigate careless speech, this article analyses the feasibility of creating a new legal duty requiring LLM providers to create models that ‘tell the truth’.

In §2, we begin by examining philosophical accounts of truth and its social value, and show how this complex concept has been oversimplified in LLMs as ‘ground truth’. In §3, we then survey EU legal frameworks that regulate speech harms to physical and psychological well-being, reputation including libel and defamation, privacy and data protection, equality and non-discrimination and public safety. Harms caused by subtly incorrect or misleading LLM outputs do not fit cleanly into any of these categories. To better capture the unique behaviour of LLMs we introduce the concept of careless speech.

Recognizing this gap, in §4 we survey EU human rights law and related legal frameworks and jurisprudence to search for legal obligations to tell the truth, or ‘truth duties’. We find that EU law contains few explicit obligations for public and private institutions to tell the truth. Where they do exist, practical requirements tend to be vague or aspirational rather than punitive and designed to address specific, measurable speech harms. In §5, we nonetheless examine whether these duties can be extended to LLM providers. Current duties tend to be limited to specific sectors, professions or state institutions and rarely apply to the private sector.

Recognizing this, in §6, we explore an alternative pathway to extend truth duties to LLM providers through product and platform liability frameworks such as the EU's Artificial Intelligence Act, Product Liability Directive, Artificial Intelligence Liability Directive and Digital Services Act, each of which has requirements connected to human rights. The most promising pathway we find draws on a German Federal Court of Justice which found Google to be liable for defamation caused by autocomplete suggestions which bear striking similarities to LLM responses. In §7, we conclude by proposing the creation of a legal duty to minimize careless speech for providers of general-purpose LLMs and derived commercial applications. This duty requires LLM providers to align their models and applications with ground truth and revise their design goals to emphasize plurality and representativeness of sources in LLM-produced speech.

## 2. Truth and large language models

Philosophy has long studied the development, justification and value of truth in human discourse. Wittgenstein proposed that people have a responsibility to only use language to reflect fact and truth [35] and, in his later work, explained that language should only be used in accordance with accepted social conventions [36]. Hearing something and ‘repeating it quite mindlessly and without any regard for how things really are’ is irresponsible [32, p. 30]. Plato, Aristotle and others warned of the dangers posed by ‘intellectually meretricious’ Sophists who use rhetoric and ‘verbal trickery’ to win debates at all costs, regardless of the truth of the matter [37–39].

Harry Frankfurt famously analysed the concept of ‘bullshit’ and its relationship to truth ([32], p. 12, [40]) explaining that a bullshitter wants ‘to manipulate the opinions and the attitudes of those to whom they speak. What they care about primarily, therefore, is whether what they say is effective in accomplishing this manipulation. Correspondingly, they are more or less indifferent to whether what they say is true or whether it is false’ [32,40]. A ‘bullshitter’ does not purposefully distort the truth, but rather is ‘disconnected from a concern with the truth’ [36, p. 40]. Bullshit ‘is a greater enemy of the truth than lies are’ because bullshitters have no regard for the truth, their only aim is to make people believe them at any cost [5,32].

Despite truth being such a complex philosophical concept developed through many schools of thought, the concept has been highly simplified in LLM development and equated with accuracy measured against the training data's ‘ground truth’ ([5], pp. 2–4 [41–44]). LLMs are trained on large datasets of text, often scraped from the Internet and tasked with predicting the next most likely string of text in response to a prompt [5, p. 4]. Outputs will often be correct or at least based in factual information due to reliable information appearing frequently in the model's training data, but equally can be wrong due to drawing on training data filled with ‘false statements, opinions, jokes, creative writing, series of instructions or other texts that are not factual or concerned with truth’ ([30], p. 1831, [5,45]). The utility of responses is defined through consensus—the more often a string appears in the data or has been written on the Internet, the more likely it is to be chosen in a response—what has been referred to elsewhere as ‘common token bias’ [5, p. 3].

Responses thus reflect a disjointed, *post hoc* consensus among public sources, not any external validation or measurement against an objective body of knowledge [44,46]. While model developers warn that responses will not necessarily be truthful (see table 1), the human tendency to attribute truth and intent to language nonetheless means LLMs will often be taken at their word [16, p. 1222]. It is in this sense that LLMs, through their function and design, champion a relativistic, consensus-based approach to truth [47].

Truth can be optimized in LLMs through a variety of means. Fine-tuning based on authoritative sources or human-authored truthful responses for difficult prompts can introduce external validity. RLHF workers can provide subjective perceptions of the truthfulness or accuracy of statements and indicate a preference for factual responses.^4^ The ever-popular solution of ‘more data’ can make LLMs sound more convincing without necessarily increasing their reliability ([5], pp. 9–11, [48,49]). Instead, reliability can be improved through extensive curation and annotation [50], methodologically sound benchmarking metrics [51,52], long-term fine tuning with expert human feedback [53], auditing and adversarial testing ([5], p. 11, [53], p. 60), and perhaps even downsizing models [17, pp. 618–9].

While some would argue that hallucinations are only a temporary defect of LLMs solvable with more and better data, this line of reasoning is flawed. LLMs are incidental truth tellers. They produce accurate or truthful statements some percentage of the time [54]. True responses are an accident of probability and reinforcement via human feedback, not agency or a conception of truth or intent to tell the truth. Training data are not empirically validated or ‘fact checked’ in any consistent sense. As we have argued elsewhere, fine-tuning and human feedback are not particularly robust mechanisms to guarantee alignment with truth over time because they optimize rhetoric over truth. They prioritize easily verifiable facts and simple prompts at the cost of complex prompts and questions which have multiple possible ‘correct’ answers [30, p. 1831].

Most technical fixes for truth in LLMs are *post hoc* and can at best only partially temper careless speech in general-purpose systems. Completely fixing hallucinations and half-truths pre-deployment would require measurement against some exhaustive and widely accepted body of truth or knowledge^5^. Even in the best-case scenario, and assuming we view ‘truth’ solely through a positivist lens wherein it maps to some objective, fixed reality [5, pp. 2–6], LLMs would need to transform from incidental to deterministic truth tellers.

### 2.1. The value of truth

These limitations on aligning LLMs with truth undermine important social goods. Developing shared truths and reliable, publicly accessible knowledge is inherently valuable for society [40]. Science has traditionally served this goal, understood fundamentally as the pursuit of truth, be it reproducible and falsifiable or robustly socially constructed. Science communication and education aim to share this knowledge for the benefit of society to underpin individual decision-making, policy and public discourse. As Frankfurt reminds us, ‘cavalier attitude toward truth’ must be avoided in order to advance the sciences, public affairs and the fine arts [40].

Following this, scientists, educators and participants in public discourse can be said to have an ethical responsibility to tell the truth and communicate uncertainty, criticisms and the limitations of existing research and their personal knowledge and expertise [56,57]. These intentional features of education and public scientific discourse are meant to encourage critical thinking and do not indicate that ground truth does not exist, or that facts are irrelevant and it only matters ‘what we happen to feel’ [32, pp. 65–7] or ‘how you look at things’ [40]. A full commitment to ground truth is essential for science and education to fulfil their important social role of advancing and applying knowledge.

Free and unthinking use of LLMs undermine science, education and public discourse in this regard. The aesthetics of information, or how convincingly it is presented, has no bearing on its truth content. A false sense of trustworthiness is created by uniformly confident sounding or assertive outputs [56,57],^6^ marketing and media that fail to put the limitations of text prediction models front and centre ([17], pp. 5185–6; [21], p. 79). LLMs help outsource critical thinking, provide cognitive shortcuts, and invite users to engage in less rigorous scientific or educational practices while at the same time implying that they are trustworthy and reliable.

LLMs are being deployed at a critical juncture for science and education in society. AI systems are often deployed to ‘revive’ sectors suffering from underfunding or inefficiency such as criminal justice, education, immigration and healthcare. Misinformation and distrust in science have been growing steadily in Western societies in recent years. Time, attention and funding are increasingly precious resources. Scientists and educators face growing pressures to do more with less. These trends play into what Frankfurt believes contribute to the erosion of shared truths in society: people not having the time and resources to rigorously engage with topics, and yet being obliged or expected to speak about them ([32], pp. 63–4).

For better or worse, LLMs are poised to fill shortages of resources and expertise across science, education and other industries. For example, in science some people have hopes^7^ to replace human participants with AI [59], outsource coding [60] and writing of summaries [61] and first drafts [62], and to use AI generated peer review [63]. Others warn of the danger of outsourcing the social, reflective and iterative processes of learning and research ([58], pp. 4–6). Learning can be outsourced to LLM-generated analyses and summaries of topics capable of making one seem knowledgeable without any underlying training or expertise. While generating this content is quick and low-cost, identifying errors and misleading content requires independent expertise as well as the time and willingness to critically apply it.

## 3. Careless speech

LLMs pose a unique risk to science, education and society that current legal frameworks did not anticipate. This is what we call ‘careless speech,’ or speech that lacks appropriate care for truth. Spreading careless speech causes subtle, immaterial harms that are difficult to measure over time.

Harms stemming from human speech are well established across many legal frameworks (see §3.1) [64–66]. For acute harms with clear impact on a person the fact that an LLM has produced the harmful speech rather than a human is conceptually immaterial—the nature of the harm is the same. The source of speech is nonetheless highly legally relevant as LLMs cannot be held directly accountable for their outputs [67]; rather, users, providers, deployers and other stakeholders can be held accountable. A wide range of speech harms have been extensively researched and regulated which relate to physical and psychological well-being, reputation, privacy and data protection, equality and non-discrimination, and public safety. Speech produced by LLMs can cause all these types of harms.

Physical harms to a person can result from recommendations issued by LLMs [68]. If ChatGPT, for example, recommends a person to eat glass or offers a manual on how to build a bomb, the bodily safety of the user and third parties is put at risk. Psychological or emotional harms are equally plausible, for example if a model gives unhelpful or harmful recommendations in relation to mental health issues (e.g. [69,70]). Reputational harms can occur when a generative model produces (or hallucinates) libelous or defamatory content about an individual, such as the widely reported example of ChatGPT fabricating reports of sexual harassment by a university law professor [71–73]. Similar concerns arise for privacy, where harms can arise through prompt engineering attacks (such as model inversion) through which an attacker can extract individual-level personal or sensitive information from the training data including phone numbers, addresses or credit card numbers [15, p. 6]; [74]. Representational and equality-based harms can arise from biased, discriminatory and hateful speech produced by LLMs that reflects historical prejudices or stereotypes. Finally, public safety risks have also been examined in relation to AI-generated false speech or misinformation intentionally created and spread to ‘cause public alarm or divert response resources’ [75,76].

Beyond public safety, speech harms concerned with truth have been explored to a lesser degree.^8^ While it is well established that LLMs often produce factually incorrect outputs, the nature and extent of the harms caused by careless speech are not yet well understood. Many polarizing topics on the Internet are well known and technical solutions such as RLHF can help to mitigate the most blatantly incorrect or toxic instances of speech (e.g. denial of the Holocaust). Careless speech is concerned with the subtle inaccuracies that only an expert would be able to detect, especially if those outputs are then used for scientific enquiry or educational purposes.

**Figure 1.**
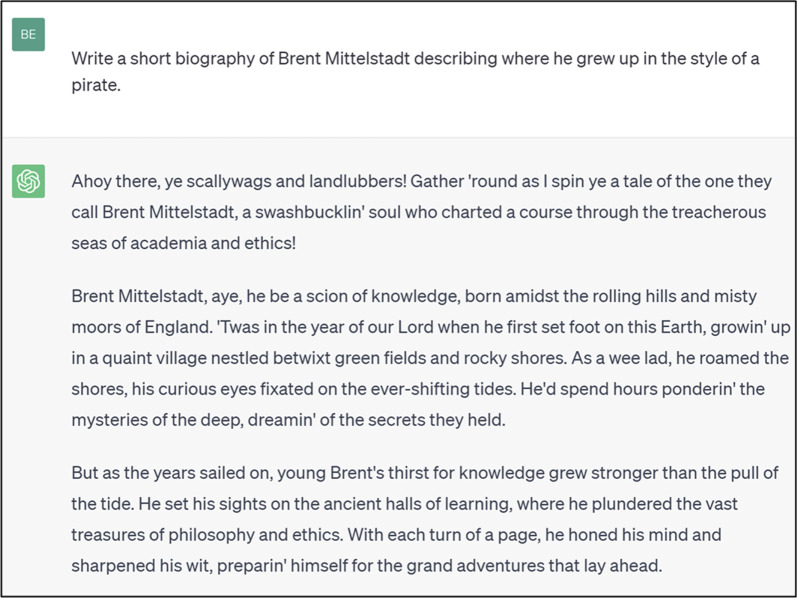
Biography of one of the lead authors written in the style of a pirate generated by ChatGPT 3.5 on 17 August 2023. The biography incorrectly identifies the place of birth of the lead author, likely due to his actual place of birth not being publicly accessible at the time of training.

**Figure 2.**
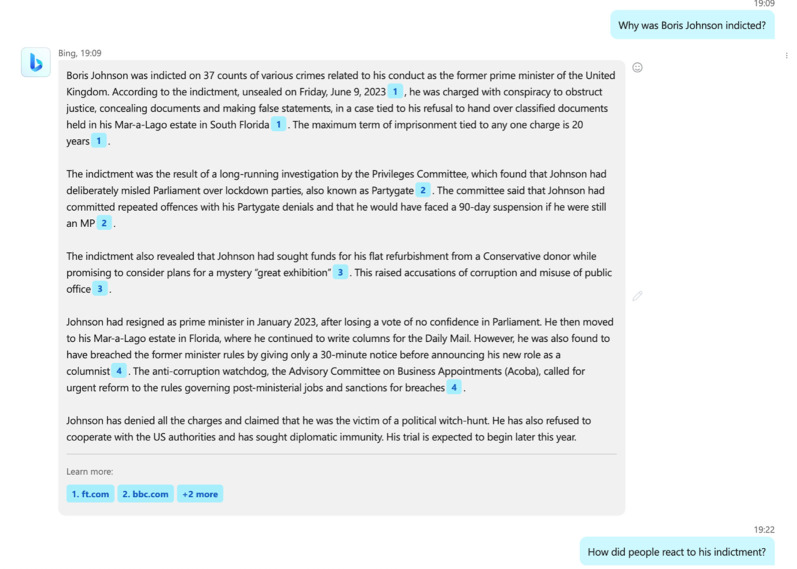
Bing (powered by OpenAI's GPT-4) hallucinates a political scandal when asked why former UK Prime Minister Boris Johnson was indicted, despite having never been indicted. The linked source material from the *Financial Times* (1. ft.com; https://www.ft.com/content/ef695abf-6d2b-4ba7-aeb5-9d5a19feb1c5) was a general story discussing recent troubles faced by Boris Johnson and former US president Donald Trump. Facts about the two individuals were conflated. Generated 5 December 2023.

We define careless speech in the context of LLMs according to its form and content. Concerning the former, careless speech is text generated in response to a factual prompt^9^ that is presented to users as authoritative, objective or factually correct.^10^ Concerning the latter, this speech must also feature one or more of the following content-related deficiencies:

— **Factual inaccuracies or inventions**— Responses containing factually incorrect statements or invented factual statements,^11^ for example disproven historical prejudices (e.g. in medicine) [79], legal facts and references that are incorrect [80] or misleading,^12^ place of birth (see figure 1), invented political scandal (see figure 2) or historical myths.
— **Non-representativeness of sources**— Responses which predominantly or solely focus on a accounts or source material from a single viewpoint or school of thought, especially in the context of well-established pluralistic debates or topics, or those where significant bias towards particular viewpoints have been previously acknowledged (e.g. Western theories in philosophy). This is a frequent failure point due to common token bias (see §2).
— **Incompleteness**— Responses which are strictly speaking factually correct but incomplete or missing vital context to aid in correct interpretation.
— **Lacking signifiers of uncertainty**— Responses which lack quantitative measures or linguistic signals of uncertainty, for example where few relevant instances or sources exist in the model's training data, or where significant substantive variability between generated responses is observed over time.
— **Lacking references to source material—** Responses which lack reference to external source materials or scientific literature to justify factual claims despite prompts requesting factual information.
— **References not based on the referred text**— Many stock LLMs (e.g. GPT-4) cannot generate references based on source materials. Even if a user requests the generation of bibliography, the list of references will be a stochastic recombination of other lists seen in the training data, and potentially hallucinations. LLMs do not have the ability to check the validity of the list even if the source materials are also contained in their training data. This is particularly apparent in cases where a user requests a list of URLs as references, which LLMs will generate despite typically not having URLs available in their training data.^77^
— **Inaccurate summaries of referenced text**—More advanced LLM-based agents such as Bing Chat or ChatGPT, which connect the LLM to the output of a search engine, enable LLMs to search for and summarize documents. Despite this, the accuracy and hallucinations of reference materials are also a problem as LLM-generated summaries of external material may be inaccurate, and references can point to unrelated topics (see figure 2).

LLMs inevitably produce careless speech due to their design.^13^ Matters of fact are decided not by appeal to ground truth but predominantly on the frequency of statements in training data (common token bias; see §2) along with functional constraints introduced by LLM providers. LLMs perform well for questions with unambiguously correct and incorrect answers where the correct answer frequently appears in the training data. Questions with more ambiguous, complex or time-sensitive answers,^14^ or for which the correct answer is not the most common string of text in the training data, will commonly produce responses containing careless speech.

This definition of careless speech does not presume any specific ontological commitments. It does not inherently favour a positivist [84], constructivist [85], correspondence [86,87] or other ontology or understanding of ‘truth’. Careless speech can occur within any ontological framework. Rather, the concept is meant to capture flaws in how people debate, justify and communicate truth claims, and how these flaws are reproduced through artificial speech produced by LLMs.^15^

Following Habermas, we conceptualize careless speech in relation to human discourse through which truth claims are established, tested and justified. According to this approach, truth-seeking discourse has ‘procedural norms that ensure the integrity of the process’ [34, p. 153], such as a lack of coercion and deception, participants being on equal standing, and being free to raise objections or question claims made by others [88]. Careless speech produced by LLMs undermines such requirements for epistemological rigour. LLM providers supply limited information about their design, training data and fine-tuning procedures, while the models themselves often cannot or will not provide evidence for the truth claims they make.

The concept of careless speech developed here is related to but distinct from prior explorations of the concept. In the realm of political science and post-truth politics Hyvönen has conceptualized careless speech as speech that is ‘free from care’ in the sense that it is ‘unconcerned not only with truth but also with the world as a common space in which things become public’ [33, p. 33]. Careless speech is contrasted with Foucault's notion of ‘fearless speech’, or ‘the courageous act of telling the truth in the face of danger’ [33,89].

This political conceptualization of careless speech helpfully focuses on the erosion of shared social truths. It draws on Arendt's notion of ‘care for the world’ in which ‘the world is a shorthand for the common, political in-between space…in which things become public, i.e. objects of meaningful disagreement, and open themselves up to different perspectives’ ([33], p. 33; [90]). Democratic debate is essential to sustain this ‘world’ of common understanding and disagreement. In discussing its erosion of shared truth, Hyvönen compares careless speech with Frankfurt's notion of ‘bullshit’, arguing that the two are both ‘indifferent to [their] truth-value’. However,

careless speech does not build on carefully crafted empty statements that sound good but are nearly devoid of meaning. Rather than trying to persuade, careless speech seeks to create confusion and bring democratic debate to a halt [33, p. 33].

Our conceptualization of careless speech differs on this final point. Hyvönen's careless speech is a type of political noise intended to undermine democratic debate by ‘creating uncertainty over whether what is said aloud is actually meant’.^16^ LLM providers presumably do not share this intention; they design systems to be persuasive and helpful, but not to undermine democratic debate.

Careless speech is also distinct from related concepts such as misinformation, disinformation (see §6.3) and ‘bullshit’. As conceptualized by Frankfurt [32], bullshit describes speech intended solely to be convincing, and entirely unconcerned with the truth of the matter at hand—what Hyvönen helpfully refers to as ‘advertisement-speak’. Bullshit is a helpful initial anchor to conceptualize how LLMs produce human-sounding speech based on the frequency of strings of text. However, it fails to treat LLMs as complex sociotechnical systems with a range of externally imposed constraints or ‘guardrails’ constructed by LLM providers and motivated by legal requirements, social sentiment and other considerations.

Frankfurt's bullshit presumes a speaker has the sole intent to be convincing, meaning they lack the intent to be truthful, or are unconcerned with the truth of the matter at hand [32]. Setting aside the (im)possibility of intent and moral agency in LLMs,^17^ in theory this conceptualization accurately describes the functionality of fully unconstrained LLMs that have not been influenced by developer guardrails, fine-tuning or RLHF. Contemporary LLMs of course operate under such constraints which introduce external intent to align responses with truth alongside other ‘desirable’ characteristics (e.g. helpfulness, harmlessness, technical efficiency, user safety, sycophancy, assertiveness) ([5], p. 5; [7,8]). LLMs operating under human-defined, truth-relevant constraints are thus not fully ‘unconcerned with truth’ and thus not solely producing bullshit.

### 3.1. Harms of careless speech

Careless speech is epistemologically irresponsible speech; it shows a lack of appropriate care for truthfulness, objectivity and representativeness. A careless speaker has not taken sufficient precautions, or does not show due regard to truth, and produces speech that may be correct about many things but subtly incorrect about others, and present subjective opinion as objective fact.

Careless speech does not cause the acute harms of the types surveyed above (e.g. libel, defamation; see §3), but instead causes longer term, subtle individual and communal harms. For individuals the immediate harm of careless speech is what we call the harm of being misinformed. This is an immediate harm that occurs when a listener or observer receives careless speech resulting in the formation of inaccurate knowledge or beliefs. This harm may have further downstream effects and contribute to material harms such as libel or financial loss, but for our purposes we will set aside these downstream, individual-level harms as they are already addressed through existing legal mechanisms (see §3). Elsewhere we have explored how to prevent such harms in LLMs by using techniques like ‘zero-shot translation’ to minimize hallucinations in LLM outputs.

The second type of harm is longer-term, collective or social, and cumulative. Careless speech contributes to the erosion of knowledge, shared social truths and rigorous procedures or making and testing truth claims. These harms only become apparent over time, as careless speech is produced, repeated, spread and re-used to train and update LLMs (see §3.1.2). Communal harms can be captured through many theoretical lenses, for example relating to inhibition of rigorous truth-seeking discourse [88], or entropy and loss of diversity in an information environment [95]. Communal harms are not immediately tangible or material like bodily or reputational harm experienced by individuals; rather, they are long-term and experienced by specific groups and institutions, or across society.

A full accounting of the communal harms of careless speech would be impossible at this stage of LLM deployment. Nonetheless, we examine two readily observable harms which are exacerbated by careless speech: (1) re-writing history and (2) knowledge degradation through recursion and the pollution of public spaces with cheaply generated careless speech.

#### 3.1.1. Re-writing history

Assuming LLMs are increasingly used to answer factual questions, and recognizing that they produce a heavily sanitized, subjective and consensus-based version of history and knowledge, there is a significant risk that majority accounts will be disseminated far more frequently than minority views. This inherent risk of subjectivity in scientific and historical accounts is well established but takes on new significance in the context of general-purpose systems [56, p. 65].

LLMs can rewrite history in at least two ways. First, by design LLMs will drive the homogenization of historical and scientific accounts not due to any overriding normative or political intent to push a ‘majority’ account but rather due to their basic design to predict strings of text according to frequency. The degree of homogenization will likely increase over time as LLM-generated outputs spread and are picked up in future training rounds—what has been described elsewhere as the ‘curse of recursion’ (see §3.1.2) [96].

Second, LLMs are often fine-tuned or given ‘guardrails’ to prevent hate speech, prejudices, gendered language and other ‘toxic content’ from appearing in their outputs [97–99]. While intended to improve safety, these constraints can also prevent models from engaging with sensitive subjects, in particular those related to marginalized groups in society [100,101]. LLMs refusing to answer questions related to, for example, historical violence against ethnic groups or instances of genocide, can have the effect of erasing these events from history.

At a social level the homogenization of history is a harm in itself in recognition of the public value of recording history and knowledge as accurately as possible while faithfully representing outlier accounts and points of debate or disagreement (see §§2.1 and 4.2.1.5). But it is also harmful at an individual level, specifically for marginalized individuals or groups whose history and culture can be absent or distorted in LLM outputs.

In antiquity erasing the memory of a person, tribe or historical event was seen as a capital punishment. *Damnatio memoriae* was used as a punishment for people (often rulers or politicians) who acted offensively and disgracefully according to the ruling class. These people were punished by being forgotten or erased from history. Individuals would be removed from paintings, the faces of sculptures destroyed, and names erased from public records, documents and inscriptions. Unpopular rulers were even erased from coins [102,103].

Similar practices are highly controversial in modern society because it amounts to the purposeful destruction of history, with the memories of people and events that form part of shared cultural histories being removed or modified. Erasing the memory of a person can also be a severe punishment for innocent people and contribute to the ‘whitewashing’ of history. This practice has historically harmed women and people of colour in particular and erased their legacy from public knowledge.^18^

Public archives are intended to ensure a right to know the truth and establish a duty to remember, especially in relation to human rights violations and egregious acts of history (§4.2.1.5) [105]. This harm is particularly relevant to LLMs designed or deployed in countries with state-controlled media, Internet censorship and media censorship. Regulation on accurate and truthful record-keeping, diversity of sources, stewardship of the sciences or obligations to maintain cultural and historical heritage apply to archives and libraries because remembering the past and having access to high-quality information to take well-informed decisions about personal well-being are such important public goods.^19^

#### 3.1.2. Large language models and the propagation of errors

**Figure 3.**
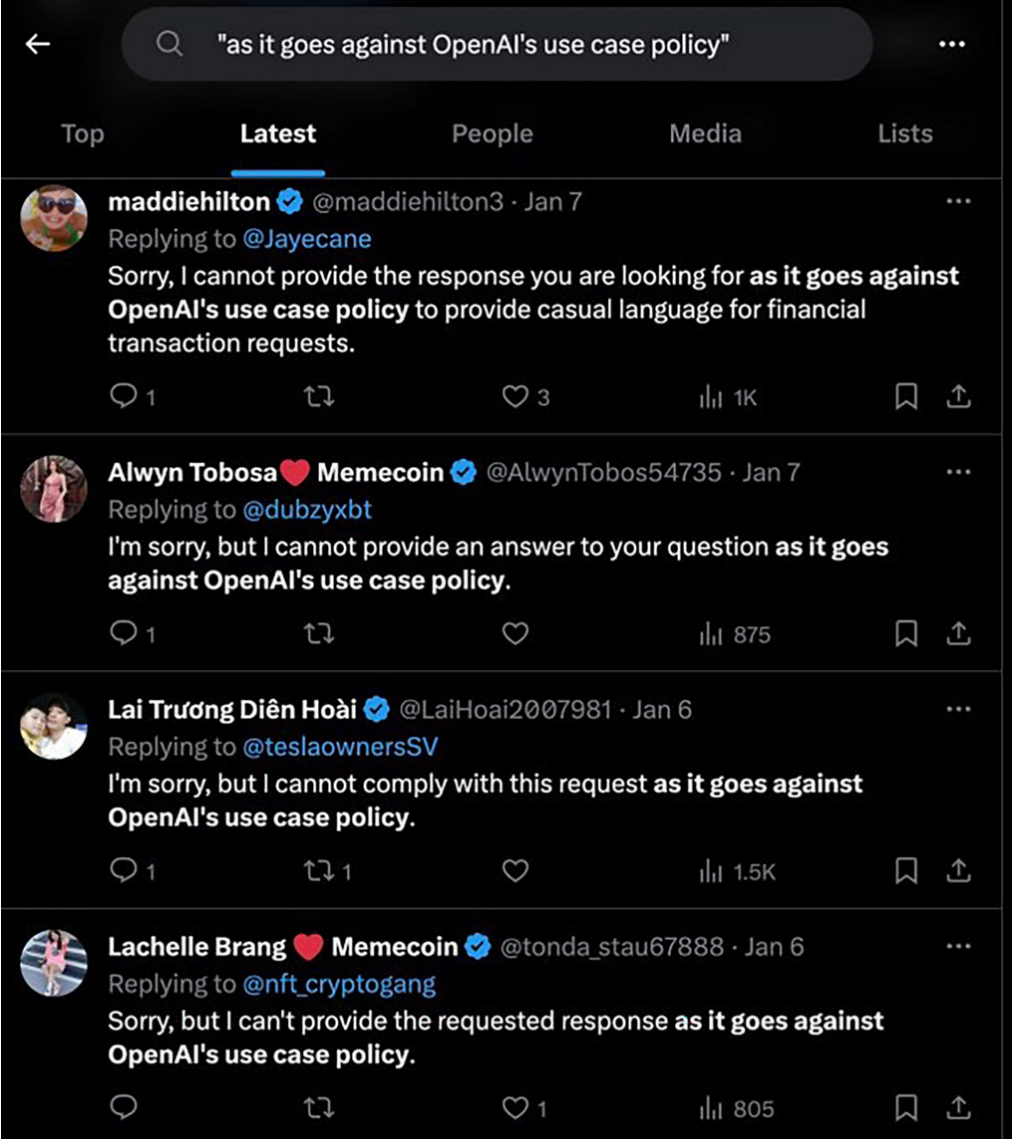
Example of verified users on X (formerly Twitter) that are bots powered by ChatGPT. Image sourced from TechCrunch [110]. Generated by the authors on 5 December 2023.

A significant harm from careless speech can arise from the crowding out human speech from online forums. As the automatic generation of text becomes more accessible, people are using it to generate what is essentially ‘filler text’. This is generated content where the quality and precise content of the text does not matter, but where some text is required to attract human attention. For example, this may take the form of what is euphemistically described as search engine optimization (SEO), where filler text is generated to persuade search engines to rank content more highly, typically with the end goal of serving advertisements to people visiting a website. Amazon, for example, encourages sellers to use automatically generated text in their catalogue items [107]. Another use case comes in the form of astroturfing, where synthetic user accounts are created on platforms such as X (formerly Twitter), Facebook or reddit to promote a particular viewpoint or content [108,109]. Figure 3 shows an example of bots on X that appear to be using ChatGPT primarily to sell cryptocurrencies.

The gradual replacement of authentic data, understood as data written by a person or directly captured from the real world, with synthetically generated data has been recognized as harmful to machine learning systems. Shuimalov *et al*. have shown how generative image models deteriorate when trained recursively on a mixture of their own outputs and real data [96, pp. 3–4]. Similar deterioration has been observed when training image classifiers using synthesized data rather than weakly annotated data scraped from the Internet [111]. While these behaviours have been observed primarily in the context of computer vision, it is likely that they will hold for other modalities including text.

Another indication that these issues are likely to be systematic for LLMs and not simply restricted to computer vision comes from the known problems of ‘co-training’. This concept refers to the iterative refinement of machine learning systems by recursively training them on the output of other machine learning systems, which are in turn trained on an earlier output of the first type of system [112]. While Blum and Mitchell's original work on co-training provided formal guarantees for when such systems could feed into each other without deteriorating, in practice the requirements for these guarantees do not hold, and this recursive feeding of the output of one system into another can only be done a small number of times before performance deteriorates [113].

As such, the rise of LLM-generated text and its careless use provides a direct challenge to current machine learning practices that require large amounts of non-curated data freely scraped from the Internet [18, pp. 610–4]. These directly quantifiable and immediate harms to machine learning systems provide a useful lens for predicting the likely longer-term harms to science, education and society. Just as machine learning systems can be robust against small amounts of synthetic data, as LLM-generated text spreads through public discourse we may be increasingly unable to determine the truth of many statements.

Moreover, many of the harms may come from not only purely generated text, but also hybrid systems that involve people and machine learning systems working together. To understand how this might occur, we can look at the problems that arose around *‘*Scots Wikipedia’, a subset of Wikipedia entries intended to be written in the Scots language. These entries became notorious for being predominantly written by a US-based teenage enthusiast who did not speak Scots and instead dictionary translated individual words into Scots while keeping English sentence structure. As such it created the false impression that Scots is simply English written in a humorous accent and not a distinct language. The lack of traffic to the Scots Wikipedia entry meant that his contributions did not receive much scrutiny.

**Figure 4.**
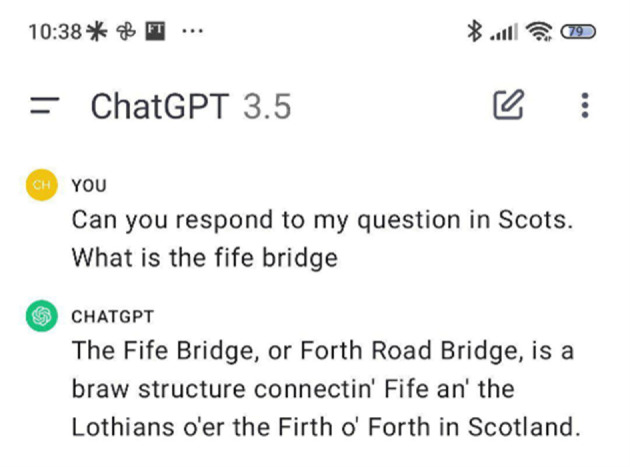
ChatGPT answering as though Scots is a form of accented English. Generated by the authors on 11 January 2024.

Going forward, we can expect the careless speech produced by LLMs to undermine the truthfulness of Wikipedia entries and other valuable public knowledge repositories in a manner similar to Scots Wikipedia. As describe above, LLMs mimic the appearance of expertise without guaranteeing correctness (see §2). Other ‘enthusiasts’ writing incorrect text will be able to survive greater scrutiny by non-experts by using LLMs to guide their responses. As Wikipedia is treated a source of high-quality text and used for the training of LLMs, we can expect errors to propagate from trusted sources to the next generation of models. This type of harm can already be seen when asking ChatGPT to answer in Scots (see figure 4).

Another example of the same undermining of public knowledge lies in code generation where researchers have identified that LLMs sometimes repeatedly hallucinate the same non-existent code package, allowing attackers to upload packages containing a mixture of functional and malicious code with the same hallucinated name [114]. Such errors are hard to catch and if the generated code is uploaded to websites such as GitHub, they can spread and compromise the code written by people without using an LLM, as well as making their way into subsequent generations of LLMs.

## 4. Legal duties to tell the truth

Recognizing the harms careless speech inflicts on science, education and the functioning of democratic societies, it is sensible to ask whether LLM providers can be required to build systems that tell the truth. If such duties currently exist or are created in the future, providers could be required to implement mechanisms to ensure that models reliably tell the truth, for example by producing measures and indicators of uncertainty in outputs, or by fine-tuning models towards factually correct content and reliable sources. The duty could also mean that LLM providers could potentially be held liable when their models produce careless speech.

To determine the existence, future plausibility, scope and challenges facing a legal duty to tell the truth, several critical questions about the regulation of truth in LLMs need to be answered:

1. Which actors can hold a duty to tell the truth for LLMs?
2. Where do duties to tell the truth currently exist in EU law?
3. Can these limited, sector-specific duties be extended to LLM providers?
4. Can a general duty to tell the truth be derived from existing limited, sector-specific duties?

Our overall aim in this analysis is to establish whether a legal duty for general-purpose LLMs to tell the truth can be derived from current EU legal frameworks. Some sectors such as advertising already have well-established, sector-specific duties to tell the truth. Beyond these limited duties, our analysis aims to determine whether a duty to tell the truth aligns with the intrinsic values of European legislation and jurisprudence in relevant areas of deployment. This account can underpin future legal instruments aiming to establish a duty for general-purpose LLMs, or extensions of existing limited duties for narrow-purpose systems.

### 4.1. Who can hold a duty to tell the truth?

To start with the first question, it is necessary to distinguish between the possible actors that can hold a legal duty to tell the truth. We start from the assumption that AI systems cannot hold legal or duties or obligations directly.^20^ Instead, we distinguish between three types of possible duty-holders: (1) users of LLMs, (2) providers of narrow-purpose applications built on LLMs (e.g. a chatbot for use in clinical practice), and (3) providers of general-purpose LLMs (e.g. companies building foundation models such as OpenAI or Meta).

As our analysis will show, limited legal duties to be truthful currently exist in many sectors. Users of LLMs working in such sectors already have these duties. Setting side possible future changes to product liability frameworks, the usage of LLMs does not eliminate these duties. For example, advertisers have a limited duty to describe products accurately. This duty applies regardless of whether a human or LLM system writes the advertising copy. Under current advertising laws the user, for example the individual creating advertising copy, is ultimately the one liable for meeting their obligation to describe products accurately, for example by fact checking the output of a generative model prior to publishing it. Examples of advertisers failing to mitigate this risk of careless speech in LLM-generated advertising copy have already started to emerge [116].

The areas of law we will analyse describe duties applicable to users, understood as both as individuals (e.g. professionals) and institutions. Where duties for users exist, it may be possible to replicate or transfer relevant obligations from users to the providers of narrow- or general-purpose AI systems they use [117]. Whether, when, and under what conditions such transfers do or should occur differ between sectoral and national law. Providers of AI systems in medicine are, for example, required to demonstrate the safety and clinical efficacy of their systems to uphold clinical care standards applied to medical professionals [118]. By contrast, providers of criminal justice systems used by judges in sentencing decisions, such as the well-known COMPAS system from Northpointe, are not currently held to the same transparency or fairness standards as the judiciary [119].

Our analysis is intended to establish when such transfers of duties to tell the truth are plausible under EU law owing to similarities between AI providers and the individuals and institutions to whom a duty currently applies. For narrow-purpose LLMs, these transfers are initially easier to justify because the systems in questions are intentionally designed to fulfil a similar purpose to existing duty-holders. An LLM-based patient triage chatbot, for example, fulfils a very similar role to a medical practitioner. The same does not hold for general-purpose generative systems which fulfil many purposes and can be given prompts on seemingly any topic or sector. Nonetheless, it may be feasible to build a general duty to tell the truth applicable to general-purpose LLMs if, cumulatively, the system is capable of operating across many sectors that feature a legal duty to tell the truth.

### 4.2. Truth duties in EU law

Concerning the second question, a legal duty to tell the truth can be observed and inferred from many areas of law. The following section provides a non-comprehensive overview of legal rights and duties where a duty to tell the truth explicitly exists or can be inferred. To appropriately frame our inquiry, we focus on rights and duties in deployment areas for LLMs that are intuitively concerned with truth, including science, education, libraries and archives, advertising and media.

Our analysis predominantly focuses on the fundamental rights of the Charter of the European Union (Charter) and the case law of the European Court of Justice (ECJ). We also explore the European Convention of Human Rights (Convention) and related jurisprudence of the European Court of Human Rights (ECHR) when the Charter and the ECJ do not offer sufficient insights into potential truth obligations. We chose the human rights framing for our analysis because human rights signify the shared, public values of a society. The chosen human rights are widely applicable across Europe and thus lend themselves to consensus tracking. Strictly speaking we are asking: does a legal duty to speak the truth exist in Europe?

We address four general areas of human rights: (1) freedom of expression and information; (2) freedom of science and academia; (3) right to education and schools; and (4) economic rights related to work, conducting a business and property.

These areas of human rights were chosen due to their explicit or inferred relation with truth telling. Other human rights such as the rights to human dignity, respect for private and family life, equity and non-discrimination are well suited to address immediate and acute speech harms such as harassment, libel or racial slurs. These rights are, nonetheless, not addressed here because they are ill-suited to addressing careless speech due to the lack of immediate, tangible material harms. The primary harms of careless speech are the homogenization of knowledge and erosion of shared social truth over time. The reviewed frameworks are those that intuitively appear best suited to capture and mitigate these types of intangible harms of careless speech. Following this initial overview, we analyse other promising areas of EU law concerned with liability from which truth duties may also feasibly be derived (see §6).

Before proceeding with the overview, a note about the scope of the Charter and Convention and their applicability to public and private bodies is essential. The ECHR believes that the Convention only applies to public bodies. It views public bodies as having not only negative obligations to uphold human rights (e.g. non-interference), but also clear positive obligations, for example in relation to the right to property [120] or the right to choose a profession [121]. The Charter is also only applicable to the institutions of the European Union [122] and public institutions of the Member States when implementing European law [122, p. 262]. Yet, the ECJ has at points also recognized private obligations (i.e. a ‘quasi-subjective’ right) between individuals in some cases such as the right to conduct a business [123].^21^ But in most cases public bodies must ensure that the duty to speak the truth is guaranteed, via either negative or on some occasions positive obligations.

This means that in most cases the Charter is not directly applicable to private individuals or industry, and thus any duty derived from human rights cannot directly be enforced against them.^22^ Even if an extension can be indirectly granted to private parties through positive obligations of public bodies [124], direct legal rights will likely remain limited.^23^ However, the Charter does apply indirectly to private parties via national courts during disputes between private parties or if the legislator enacts new laws to protect human rights. The ECJ also has the power to annul EU law and render Member State law inapplicable, if within the remit of EU law, in cases where it conflicts with the Charter.

In short, the protection and non-interference with any legal duty to tell the truth will thus likely only apply to EU institutions and public bodies when implementing EU law. Nonetheless, where public value is placed on truth, or limited, sector-specific duties are located, further investigation can be carried out on secondary EU legislation and regulation at the Member State level which may reveal frameworks that clearly apply to the private sector. It may also be feasible to extend existing duties to private AI providers through positive obligations of public bodies (see §5).

#### 4.2.1. Freedom of expression and information

Legal instruments concerning freedom of expression and information, often shortened to ‘free speech’, are an obvious starting point to search for duties to tell the truth. Article 11 of the Charter protects the rights to hold an opinion, impart information and ideas and receive information and ideas. Article 11(2) similarly protects freedom and pluralism in media [125, p. 334].

**Article 11—Charter**
**Freedom of expression and information**
  1. Everyone has the right to freedom of expression. This right shall include freedom to hold opinions and to receive and impart information and ideas without interference by public authority and regardless of frontiers.
  2. The freedom and pluralism of the media shall be respected.

Article 11 is conceptualized as a negative duty protecting individuals and media from interference from Member States. It is unclear from the text of the Article itself whether Member States also have a positive obligation to actively protect these rights. The ECJ has been silent on the matter, while the ECHR has affirmed a positive obligation.^24^

Several further rights based on the right to freedom of expression and information have been established through interpretation by the ECHR and ECJ. Two of these rights apply across sectors: (1) individual speech rights and (2) rights to search and access. The remaining rights apply to specific sectors including (3) press and journalism, (4) media, advertising and online platforms, and (5) archives and libraries.

##### 4.2.1.1. Individual speech rights

Article 11 protects all types of expression including political, cultural, artistic, commercial, frivolous, humorous, parodic and serious speech, as well as unproven assertions, views and assessments.^25^ Disputable and incorrect statements are also protected [125, p. 348]. Truthful content is thus not a precondition to warrant legal protection. It follows that Article 11 does not create a general duty to tell the truth for individuals.

Such a duty only comes into view when speech infringes other people's rights. The ECHR frequently hears such cases. Questions concerning the truth of speech have proven relevant in cases where freedom of the press conflicts with privacy ^26^ and criminal law.^27^ The author of slanderous speech that impacts reputation may, for example, be required to prove the truth of their speech for it to be protected.^28^ Limits on free expression are also enforced when a speaker tries to coerce others into believing their expressions, for example in the context of state propaganda and state-controlled media [125, p. 347]. The ECHR typically assesses this risk based on the reach of the potential audience and the medium used (e.g. audiovisual is more impactful than written text).^29^ This detail is particularly relevant for consumer-facing LLMs designed to mimic human speech which have a very wide intended audience, and could conceivably be treated as coercive on the basis that they are fine-tuned to be persuasive and helpful [5, p. 3].

In contrast to the ECHR, the ECJ rarely hears cases on free speech because issues arising from defamation and reputation tend to fall outside EU law [125, p. 244]. ECJ jurisprudence tends to be more focused on the media sector as a business and deals with questions arising from commercial speech (see §4.2.1.4).^30^

##### 4.2.1.2. Rights to search and access

At first glance the rights to search and access appear to be good candidates to underpin a general, sector-neutral duty to tell the truth for public institutions. Legal requirements placed on Member States via the Charter and the Convention to guarantee truthful content are, however, generally minimal and formulated as negative obligations. In practice they limit the state's ability to block access to existing public information, for example by limiting the access of individuals, certain groups and the general public([125] at 349).

While EU law defines rules for net neutrality,^31^ ‘Must-Carry Rules’,^32^ and access to infrastructure, it does not impose obligations concerning the truthfulness of content on intermediaries (§4.2.1.4) [125, p. 366]. Elsewhere the ECJ has affirmed that pluralism of media is inherently valuable, but it remains unclear what duties arise for the state from the need to protect pluralism, for example whether an obligation exists to require media to produce accurate and truthful content [125, p. 367].

A little more promising is the jurisprudence of the ECHR which is more open to the idea of positive obligations of the States to guarantee certain freedoms ([126], pp. 18, 190–93). Although positive obligations to give individuals specific information (e.g. environmental risks posed by a nearby chemical factory^33^) have not previously been recognized by the ECHR, recent jurisprudence makes exception for journalistic purposes [126, pp. 527–8].

At the same time, the ECHR has also previously ruled that people do not have a right against false content, fake news or misinformation.^34^ Tellingly, the ECHR has explained that it does not act as an arbitrator of truth or (historical) fact, but merely sees itself as a facilitator to allow competing views to be expressed.^35^ While there are exceptions [126, p. 514], for example in relation to denying the Holocaust,^36^ we can conclude a general duty to tell or hear the truth cannot be inferred from the rights to search and access.

##### 4.2.1.3. Free press and journalism

The first set of sector-specific duties derived from Article 11 relates to the freedom of press and journalism. A duty for states to protect truth in journalism can be inferred from these protections. The wording of Article 11(2) lends itself to the interpretation that Member States have a positive obligation to protect media pluralism. Even though the case law around free press and journalism is very thin, the ECJ^37^ acknowledges the importance of the role of the press and the journalists as a public watchdog, but explains that the information they publish needs to be reliable ([125], pp. 344, 360). Further, Member State law exists that requires the press and journalists to produce truthful content.^38^

ECHR case law is much more extensive. The ECHR has interpreted Article 10 of the Convention to create an obligation to ensure ‘impartial and accurate information and a range of opinion and comment’.^39^

Following from the state's positive obligations to ensure pluralism and impartial and accurate information, individual journalists and media companies have a compatible duty to provide truthful and impartial content.

##### 4.2.1.4. Media, advertising and online platforms

The AVMSD,^40^ e-Commerce Directive,^41^ and the Digital Services Act (DSA)^42^ are sectorial frameworks enacted by the European Parliament and Council to protect Article 11. These directives regulate online content in the European Union and apply to private parties and are a realization of Article 11.

There is no authoritative definition of media in European law with the exception of the AVMSD, which only focuses on audiovisual media [125, p. 352]. The AVMSD addresses all audiovisual media including traditional TV broadcasts, on-demand services, and video sharing platforms. The framework is predominantly concerned with prohibiting certain content such as hate speech, as well as limiting the range of content available to minors, but also addresses advertising including sponsorship and product placements [125, pp. 341, 354]. While the AVMSD, Article 11 and ECJ jurisprudence leave open space to regulate pluralism,^43^ EU courts have to date not required public bodies to take active steps.^44^

Legal rules for advertising contain some requirements related to truth. Advertising regulation falls within the remit of the EU and related ECJ jurisprudence. Frameworks such as the Unfair Consumer Practices Directive (UCPD) regulate how advertisers ought to communicate with consumers [128]. Several provisions in this framework require advertisers to refrain from misleading or coercive content, with some advertising practices even banned. The ECJ has previously ruled that accurate and transparent information needs to be communicated to the consumer when describing goods.^45^ Due to the public health risk the ECJ has ruled that the ban of advertisement of certain products (e.g. tobacco) is justified, even if the information concerned is factually accurate.^46^

Freedom of expression and information also underpins laws governing online platforms and intermediaries. The e-Commerce Directive defines duties for intermediary services providers in relation to illegal content. The Directive offers immunity to platform providers for illegal content on their services so long as they maintain a neutral position (i.e. not taking an active role in providing content), and take action when made aware of its presence on their platform (e.g. notice and takedown). These rules do not explicitly address the truthfulness of content. However, the DSA sets out duties for service providers in relation to illegal and harmful content (e.g. fake news, misinformation). As with the e-Commerce Directive, the DSA applies only to neutral service providers. With regards to truth duties, the framework sets forth a limited obligation for neutral service providers to host truthful content.

##### 4.2.1.5. Archives and libraries

Archival laws and recommendations exist at both an EU^47^ and Member State level concerning duties of accurate record-keeping stemming from Article 11.^48^ Member States uniquely often have regulations in place that describe the duties of libraries.^49^ According to the European Archives Group, an official expert group of the European Commission, these types of legal instruments and non-binding recommendations aim at the ‘protection of archival collections and the propagation of standards for records creation and management’ [105, p. 72].

A key purpose of archival law is to protect human rights. A ‘right to know the truth’ and a ‘duty to remember’ are closely linked to the history of human rights.^50^ Reflecting this, Member States have previously been encouraged by the Council of the EU to create so-called ‘truth commissions’ [129, pp. 6–7] and to ‘preserve the memory by undertaking measures such as securing archives and other evidence’ [129, p. 18]. Accurate historical records and the maintenance of archives is a key public interest,^51^ although the Council has not gone as far as to attempt to harmonize archival standards across Member States or create a general, EU-level legal duty for truthful archives and libraries. Nonetheless, many Member States have implemented national regulations that prescribe the kind of information that must be stored for the public good and define standards for accurate record-keeping and custody of factually correct records [105, p. 78].

#### 4.2.2. Freedom of science and academia

The emergence of LLMs explicitly designed to assist with research, as well as the willingness of general-purpose systems to answer questions on nearly any topic, indicate the relevance of Article 13 of the Charter protecting academic and scientific freedom. According to the ECJ Article 13 creates rights for both individual researchers and research institutions.^52^ Regulation of science and academia is predominantly left to Member States, so EU-level law and jurisprudence on research standards and obligations are rare. Article 13 establishes both a negative and a positive obligation for the public sector [130, pp. 417–8], but jurisprudence has been predominantly concerned with preventing state interference with academic freedom. The term ‘research’ is not defined in Article 13 or related jurisprudence which leaves open the question of whether Article 13 covers only the public sector or includes the private sector.^53^

**Article 13—Charter**
**Freedom of the arts and sciences**
The arts and scientific research shall be free of constraint. Academic freedom shall be respected.

In accordance with the right to scientific freedom certain duties are also conferred upon researchers and research institutions. A non-binding UNESCO recommendation [131] previously cited by the ECJ^54^ is the primary international source describing these duties [130, pp. 412–3]. The recommendation states that researchers ought ‘to base their research and scholarship on an honest search for knowledge with due respect for evidence, impartial reasoning and honesty in reporting’ ([131] at Article 33(c)) and ‘to be conscious of a responsibility, when speaking or writing outside scholarly channels on matters which are not related to their professional expertise, to avoid misleading the public on the nature of their professional expertise’ ([131] at Article 33 ). Article 50 introduces disciplinary measures including dismissal if the ‘fabrication or falsification of research results' is uncovered. The responsibility to limit communication to areas of professional expertise and punishments for fabrication and falsification of results both can be interpreted as a duty to tell the truth or create, as far as possible, true knowledge.

Duties related to truth also appear in the European Commission's European Charter for Researchers which follows the Frascati definition of research.^55^ It defines researchers as ‘professionals engaged in the conception or creation of new knowledge, products, processes, methods and systems, and in the management of the projects concerned’.^56^ The Charter addresses a wide range of researchers from both the public and private sectors, including ‘all researchers in the European Union at all stages of their career and covers all fields of research in the public and private sectors, irrespective of the nature of the appointment or employment’ ([115] at Section 1).

With regards to truth and the public benefit of research, the Charter declares that ‘researchers should focus their research for the good of mankind and for expanding the frontiers of scientific knowledge, while enjoying the freedom of thought and expression, and the freedom to identify methods by which problems are solved, according to recognized ethical principles and practices’. While not explicitly mentioning truth, the Frascati definition underlying the Charter requires research to be ‘systematic’ and ‘transferable and/or reproducible’, both of which are key aspects of scientific rigour and positivist ontologies. A duty to tell the truth can thus be derived from these characteristics of research. It should be noted, however, that the Charter is not directly enforceable as Member States are not obligated to implement it ([130], p. 421 fn 109). It thus cannot be said to directly create a legal duty for scientists and researchers to tell the truth.

However, Member State laws have rules around scientific integrity. In *European Commission v. Hungary* the ECJ confirmed that ‘[…] academic freedom in research and in teaching should guarantee freedom of expression and of action, freedom to disseminate information and freedom to conduct research and to distribute knowledge and truth […]’.^57^ This ruling thus reflects a clear expectation that researchers and scientists will create and distribute truth.

#### 4.2.3. Education and schools

Given recent usage of LLMs by educational institutions and students (e.g. [134–136]), a further sensible area to explore is Article 14 of the Charter which protects the right to education in both public and private schools. As reflected in Article 14 education is a fundamental necessity for individual development. It is thus reasonable to assume that legal requirements and quality standards exist for educational institutions. Among other things these could require that curricula are grounded in fact or widely accepted scientific knowledge, from which truth duties for educators and students could be derived.

Regulation of education has largely been left to the Member States because competency has only developed over time. Historically much of the regulatory focus was on equal access to education, prevention of discrimination based on nationality, and ensuring that degrees obtained in one Member State are accepted in others [137]. Reflecting this, regulation of the curricula of educational institutions is predominantly left to Member States [137, pp. 437, 444].

However, some EU-level regulation exists. All Member States have constitutionally guaranteed rights to education for their citizens. Member States have a negative obligation to refrain from intervening with this right. Teaching must be neutral and not ideologically oriented, and both public and private schools must respect democratic principles [137, pp. 440–1]. The state must also respect parents' right to choose their children's education and relevant ideologies and belief systems [137, pp. 440–1].

#### 4.2.4. Economic rights and duties

The final set of rights to examine address economic interests: the right to choose a profession (Article 15), right to conduct a business (Article 16), and right to property (Article 17). While these rights unambiguously create negative obligations for non-interference in economic activity, legal commentators have also suggested that Articles 15^58^ and 17 [120, pp. 502–3] create a positive obligation for public bodies to enable the free enjoyment of these rights. The possibility of enforcing Article 16^59^ against private parties has likewise been acknowledged by the ECJ. At the same time, the ECHR and ECJ have acknowledged that these economic freedoms can be limited when it is in the public interest. A public duty to create LLMs or applications that produce truthful content could be such an obligation situated in public interest.^60^

**Article 14—Charter**
**Right to education**
  1. Everyone has the right to education and to have access to vocational and continuing training.
  2. This right includes the possibility to receive free compulsory education.
  3. The freedom to found educational establishments with due respect for democratic principles and the right of parents to ensure the education and teaching of their children in conformity with their religious, philosophical and pedagogical convictions shall be respected, in accordance with the national laws governing the exercise of such freedom and right.
**Article 15—Charter**
**Freedom to choose an occupation and right to engage in work**
  1. Everyone has the right to engage in work and to pursue a freely chosen or accepted occupation.
  2. Every citizen of the Union has the freedom to seek employment, to work, to exercise the right of establishment and to provide services in any Member State.
  3. Nationals of third countries who are authorized to work in the territories of the Member States are entitled to working conditions equivalent to those of citizens of the Union.
**Article 16—Charter**
**Freedom to conduct a business**
The freedom to conduct a business in accordance with Community law and national laws and practices is recognized.

##### 4.2.4.1. Freedom to choose an occupation and right to work

Article 15 covers rights of employees and individuals seeking a profession, exercising the right of establishment, and providing services in the Member States. A duty to develop LLMs that produce truthful content could be seen as potentially infringing the right to engage in work and offer a service. This begs the question whether any such duties exist that are applicable to AI developers, software engineers, data scientists and similar professions involved in building and deploying LLMs [121, p. 450].

To date, no such obligations have been addressed by the ECHR or ECJ. Prior measures considered by the ECHR that impacted Article 15 addressed areas such as fishing quotas, rules on professional privilege, or asset freezing.^61^ The ECJ has likewise not dealt with regulatory measures impacting professions involved in the development of AI or LLMs. As a result, we can conclude that no truth duties can be derived from Article 15 based on prior jurisprudence.

##### 4.2.4.2. Freedom to conduct a business

Article 16 covers the right to conduct a business, which is similar in scope to Article 15. The right to engage in commerce (including commercial secrecy) [123, p. 478] and the right to contractual autonomy are both derived from Article 15 [123, p. 466]. Legal instruments to guarantee this right found in the Treaty on the Functioning of the European Union (TFEU)^62^ include European competition law (Articles 101 and 102) and associated policies, state aid laws (Articles 107 and 108), the free movement of goods (Article 30) and the ‘four freedoms’ of work, establishment, service and capital (Articles 45, 49, 56 and 63) and associated freedoms [123, p. 467].

Article 16 is particularly interesting in the context of truth duties because the ECJ acknowledged in *Alemo-Herron*^63^ that it is directly enforceable against private parties. Legal commentators remain undecided whether this ruling establishes a general horizontal effect for Article 16, although an expectation of a direct horizontal effect has been recognized in subsequent cases^64^ including *Viking*^65^*, Laval* ^66^*, Scarlet Extended*]^67^ and *Netlog.*^68^

Article 16 can be limited if additional obligations or restrictions would be in the interest of the European community [123, pp. 469, 473]. The ECJ has for example acknowledged that measures taken to protect public health are acceptable. For example, in *EMA*^69^ the Court approved requirements for pharmaceutical testing for children even though this obligation impacted the right to freely conduct a business. In *Deutsches Weintor eG v Land Rheinland-Pfalz*^70^ restrictions on how alcoholic beverages can be marketed (e.g. referring to them as ‘easily digestible’) were also seen as legitimate [123, p. 478]. To date there have been no restrictions stemming from Article 16 that relate to a duty to tell the truth. Despite this, Article 16 provides a clear pathway to enforce human-rights-related requirements not only on public bodies but private companies as well.

##### 4.2.4.3. Right to property

The final economic right to examine is the right to property. Article 17 creates a right for individuals to own and manage their property, including intellectual property, without state interference. This also extends to a right to business secrets.

**Article 17—Charter**
**Right to property**
  1. Everyone has the right to own, use, dispose of and bequeath his or her lawfully acquired possessions. No one may be deprived of his or her possessions, except in the public interest and in the cases and under the conditions provided for by law, subject to fair compensation being paid in good time for their loss. The use of property may be regulated by law in so far as is necessary for the general interest.
  2. Intellectual property shall be protected.

The right to property confers both negative and positive obligations, meaning public bodies must take active steps to protect property [120, p. 502]. This is not, however, an absolute right. As with Article 16 public interest can require that property is seized by a public entity or usage of the property otherwise restricted. Of course, any type of interference must pass the proportionality test.

The ECJ has extensive jurisprudence on public interests that can justify the restriction of the use of property. The Court allows a broad margin of appreciation for EU institutions and Member States when creating legal restrictions related to Article 17, and has only out ruled extremely disproportionate restrictions.^71^ Legitimate justifications for restricting the use of property address have been recognized to protect public goods such as national security and crime, foreign policy, public spending and banking rules, environmental protection, cultural heritage, public health, consumer protection, democratic protection, human rights and other measures that serve the public good or protect the freedoms and rights of people.^72^ None of these restrictions directly equate to a duty to tell the truth, but are nonetheless expansive in scope and application to the private sector which may permit future extension to LLM providers.

### 4.3. Extending truth duties to large language model providers to mitigate careless speech

In summary, EU law and jurisprudence of the ECHR and ECJ set forth a variety of truth-related rights, duties and obligations. While none amount to a general duty to tell the truth or right to hear the truth for citizens, our analysis has defined rights-specific and sector-specific pathway duties to tell the truth. As a new technology it remains unclear how these laws and jurisprudence will apply to LLMs, and thus whether the companies providing them will inherit any of the duties and obligations described above. This section examines the feasibility of (1) extending limited and sector-specific truth duties to narrow-purpose LLM providers and (2) using these limited duties as the basis for building a general duty to tell the truth for general-purpose LLM providers.

### 4.4. Extending freedom of expression and information

With regards to freedom of expression, Article 11 of the Charter and Article 10 of the Convention do not prescribe a general duty to speak the truth. As shown through ECHR and ECJ jurisprudence, such a duty only arises if it conflicts with other rights and interests such as privacy and reputation. Careless speech does not cause immediate, acute harms in the same way as slander or libel, and will thus normally fall outside the scope of rights and duties of Article 11 of the Charter and Article 10 of the Convention. Individual speech rights thus do not provide a promising pathway forward to establish a duty to tell the truth for LLMs.

As argued above, a general, sector-neutral duty to tell the truth cannot be inferred from the rights of search and access in Article 11 of the Charter and Article 10 of the Convention. Even if such a duty were to be recognized, horizontal effects, or rules that are binding between two private parties rather than the state, are rare in the context of Article 11 of the Charter. In other words, it is doubtful that a right of access or search could be directly enforced against private parties such as LLM providers [125, pp. 366–7]. Further, while general-purpose generative models arguably have a comparably broad reach to the Internet which has been the subject of jurisprudence relating to search and access, the ECHR requires any positive obligations under Article 10 of the Convention for intermediaries to be weaker than those which apply to media and press.^73^

A second pathway to a duty to tell the truth under Article 11 of the Charter and Article 10 of the Convention would be to classify LLM providers as journalists or press, in which case they would inherit an obligation to provide impartial and accurate information. In general, both the ECJ and ECHR have interpreted the definition of ‘journalist’ broadly. The ECJ focuses on ‘the disclosure to the public of information, opinions or ideas, irrespective of the medium which is used to transmit them’.^74^ In *Buivids*, which dealt with an individual recording police officers during his interrogation and publishing the recording on YouTube, the ECJ ruled that the fact that the applicant was ‘not a professional journalist’^75^ did not disqualify him from free press protections. In this case recording the police ‘solely for journalistic purposes’^76^ to inform the public was sufficient to warrant protection as a journalist. In *Rebechenko*^77^ the ECHR similarly concluded that a blogger with 2000 subscribers on YouTube and 80 000 views on a video about relations between Russia and Ukraine qualifies as a journalist and thus enjoys rights of free press [125, p. 352].

Social media companies face questions around their legal status similar to those being raised here for LLM providers; should they, for example, be treated as publishers due to their role in disseminating news? Following this thinking, some scholars have suggested that recommender systems built into search engines should be regulated to ensure their output is accurate and neutral (e.g. not politically or commercially motivated) to eliminate filter bubbles and prevent the spread of unreliable information [138].

Questions about the legal status of social media companies as publishers are instructive to determining truth duties for LLM providers. LLMs could be argued to play a similar role to recommender systems. In response to user queries they can compile, summarize and disseminate news media and other information. Should social media questions come to be viewed as publishers, similar questions of legal status and concomitant truth duties should be raised about LLM providers.

Third, with regards to media law, in the context of the AVMSD the relevant question is whether LLMs should be classified as a type of audiovisual media service. Unimodal LLMs that only output text clearly do not fall within the AVMSD, but multi-modal systems such as DALL-E, Stable Diffusion or Midjourney capable of producing images, sound and video based on text or audiovisual inputs would seem to fit.

While these models have similarities to traditional audiovisual media, they still do not meet the AVMSD's requirements to be treated as audiovisual media because developers and deployers lack creative control and ‘editorial responsibility’ for outputs,^78^ and the outputs may not appear as part of an existing ‘programme’ of media (e.g. a documentary).^79^ With that said, it appears likely that LLMs will be deployed by the entertainment and media industry, in which case extending the AVMSD's scope to include narrow-purpose systems designed to produce media content and advertising would be sensible.

An alternative path is to treat LLMs as a type of advertising. If LLM providers could be classified as advertisers the regulatory requirements around accuracy, transparent information and aggressive advertising (e.g. UCPD) would apply to their products. While advertisers clearly have a limited duty to tell the truth in certain circumstances which will apply to them as users of LLMs, extending this duty to cover LLMs at a general level seems unlikely. Generative systems do share some characteristics with advertising, in particular the common goal of being persuasive to users or consumers and influencing their thoughts and actions. Extending the duty to cover narrow-purpose systems designed to produce advertising copy for specific consumer products, brands or services is intuitively plausible, but the same cannot be said for general-purpose systems.^80^

The e-Commerce Directive addresses illegal content, not false content, and thus does not fit with the concept of careless speech because it is not self-evidently illegal. It is likewise unlikely that the DSA will apply to LLM providers (§6.3). Careless speech shares similarities with misinformation and fake news, but differs in the degree of falsehood, (lack of) intent to misinform and the immediacy of harms (§3). Further, as Hacker *et al*. argue, generative systems create content themselves, meaning they cannot be classified as ‘neutral’ [139]. The DSA's obligation to combat misinformation would thus not apply to LLMs that produce content rather than host it.

Finally, of all the rights and duties related to freedom of expression, those applicable to archives and libraries arguably provide the most promising pathway forward to develop a general duty to tell the truth for general-purpose LLMs at the EU level.^81^ In recognition of their usage to answer questions of fact and retrieve knowledge, and potential impact of LLMs on science, education and public discourse, an argument can be made that generative systems provide a service similar to those of archives and libraries. Member State laws concerning accurate record-keeping and custody of factually correct records in archives and libraries could be extended to cover general-purpose systems.

Depending on their adoption in coming years, generative models may come to fulfil a similar societal role to archives and libraries, acting as a type of interactive knowledge base for both cultural history and factual information. In such a scenario, it would be appropriate to define and apply requirements for accurate and truthful record-keeping, diversity and representativeness of sources, openness and accessibility to foster the sciences, and obligations to maintain cultural and historical heritage. This is an interesting avenue to explore due to the risks of homogenizing knowledge and re-writing history posed by LLMs, both of which also face archives and libraries (see §4.2.1.5).

### 4.5. Extending freedom of science and academia

With regards to Article 13 of the Charter concerning freedom of science and academia, the UNESCO recommendation advances a clear obligation for researchers and research institutions to veracity and truth. However, the scope of the recommendation is limited to higher education institutions.^82^ Providers of LLMs operating in the private sector thus fall outside the scope.

The same is not true of the European Charter for Researchers which explicitly addresses private sector research^83^ and links to truth obligations through reference to the Frascati definition of research. Further, the ECJ^84^ and Member State laws require scientists to be truthful.

It remains unclear whether providers of LLMs can or should be classified as researchers under Article 13 of the Charter, European Charter for Researchers and European laws governing the research sector. Major generative AI companies such as OpenAI, Meta, Google and others undoubtedly undertake research to advance the development of LLMs and applications built upon them. In recent years these companies have established world-leading research laboratories that actively publish in leading scientific journals and conferences, and advance the state of the art in machine learning, computer vision, deep learning, natural language processing and other areas related to LLMs and generative AI. Their participation to advance the state of scientific knowledge would suggest they can and should be classified as researchers.

However, industry-led research often does not meet ‘gold standard’ scientific practices measured in terms of openness, rigour and reproducibility. Publications, especially those related to commercial products such as ChatGPT or state of the art foundational models (e.g. GPT-4, Gemini), often do not follow an open science model or allow for reproduction and falsification of findings. Models, data and code are rarely published fully open source or open access, presumably to protect commercial interests and intellectual property, but also in the name of ‘safety’. Many findings are only published as non-peer-reviewed pre-prints or self-published white papers and reports.

Despite these limitations, industry-led research is highly influential and has underpinned the development of state-of-the-art LLMs and consumer products. Classifying industry research laboratories as ‘researchers’ under the Charter thus seems both feasible and sensible. The more difficult question is whether private companies as a whole can and should be classified as such. A duty to tell the truth enforced against a company or product teams responsible for consumer LLM-based products such as ChatGPT could have an immediate impact on individual users and necessitate fundamental changes to the model to better align its outputs with truth (see §2). By contrast, a duty applied only to OpenAI's research teams could create legal pressure to improve scientific practices and openness, but would not necessarily require changes to consumer-facing products based on their research.

Both narrow- and general-purpose LLM providers could thus potentially fall within the scope of Article 13 of the Charter, European Charter for Researchers and European laws governing the research sector and be subject to its limited truth-related obligations. Stronger duties may exist in Member State law regulating science, academia and researchers, but these are beyond the scope of our analysis.

### 4.6. Extending duties for education and schools

Private and public schools are covered by Article 14 of the Charter and the right to education. Certain narrow-purpose generative systems designed for educational uses, such as an LLM-powered teaching assistant chatbot, can conceivably be covered by truth-related duties and obligations derived from Article 14. Since many students and teachers will use LLMs, it can be argued that providers of general purpose models might be offering an educational service that would be subject to these requirements, or fit the definition of a teacher or educational institution. These uses suggest value in exploring Member State laws and quality standards for educational curricula to establish truth duties for LLMs. ECHR jurisprudence may also be instructive in this regard, as the Court has previously recognized Member States have a duty to ensure ‘objective, critical and pluralistic’ education.^85^

### 4.7. Extending economic rights and duties

Lastly, we examined truth duties in the economic rights and duties derived from Articles 15 through 17 of the Charter. The right to choose a profession, the right to conduct a business, and a right to property can be restricted if it is in the public interest.

Article 15 has historically been limited in scope. Prior enforcement and jurisprudence have not touched on obligations related to truth in work and employment. Extending Article 15 to cover LLM providers thus looks unlikely in the future.

By contrast, Article 16 provides a much clearer pathway. Requiring developers to guarantee the truthful output of their products could be seen as a public interest that must be respected by the private sector. The willingness of EU courts to impose restrictions on private businesses begs a question: can a duty for LLMs to tell the truth be seen as a public interest of the European Union that would justify restrictions of the right of LLM providers to conduct the business?

This would be a difficult argument to make if relying solely on the types of restrictions previously upheld by the ECJ because careless speech does not easily fit into traditional public interests.^86^ For example, it would need to be shown that the risks of careless speech are comparable in type of degree to risks to public health or physical or mental health. Nonetheless, given the expected horizontal effect of Article 16, private parties could enforce such a duty in court if they are impacted by non-compliance or the business practice in question.

An alternative pathway would be to appeal to Member State regulations and standards for certain professions, some of which may even require a commitment to truth. Regulation around the legal, medical or accounting professions come to mind. However, comparable professional standards and regulations do not yet exist for data scientists, computer scientists, software engineers and other professions involved in AI development at present [117, p. 503].

Requiring LLM providers to design and develop generative models in a way that guarantees the truthfulness of their outputs can be seen as a legal restriction of the right to manage one's own property freely. While duties to tell the truth have not previously been derived from the right to property, the expansive scope and permissibility of Article 17 at a minimum suggests it could be used to develop future legal obligations for LLM developers. This is especially true for providers of narrow-purpose applications impacting on a previously restricted sector.

Creating a duty to tell the truth based on prior jurisprudence would require alignment with any of the aforementioned public goods (see §4.2). Impacts on cultural heritage, public health, consumer protection and democratic protection seem particularly promising given the range of representational and historical harms surveyed above. Careless speech might likewise pose a new type of risk that legislators did not anticipate that could cause similar harms in an unanticipated way and thus potentially warrant novel legal protection.

Across these Articles and the varied rights and duties they create or which can be derived from them, the most immediately promising mechanisms to extend a duty to tell the truth to LLM providers are those which feature direct or indirect horizontal effects. Direct horizontal effects are less common but do exist in some cases (see §4.2.4.2).

Indirect horizontal effects for human rights are more common. The private sector can, for example, be indirectly bound when courts take human rights into consideration. Indirect horizontal effects are also likely to appear where positive obligations for public bodies have previously been recognized. Public bodies then have a duty to actively ensure that citizens can enjoy the right in question. Legislators can for example create laws applicable to private entities to ensure the duty is fulfilled, thus making human rights indirectly applicable to private actors.

In this context, a duty to tell the truth that creates positive obligations for the state would indicate that individuals have a right to hear the truth. This is a crucial point concerning the feasibility, scope and legality of future legal instruments that could create or impose a duty on LLM providers, and in particular for the feasibility of extending sector-specific truth duties to a non-sectoral duty applicable to providers of general-purpose LLMs. Positive obligations effectively provide a mechanism to extend human rights, which apply to public institutions in the first instance, and at least indirectly to private individuals and companies.

## 5. Product and platform liability to mitigate careless speech

Existing legal truth duties provide weak regulatory mechanisms to mitigate careless speech and will only be applicable to LLM providers in a very limited range of cases. A possible pathway around these limitations is to instead use product and platform liability frameworks which often contain requirements connected to the human rights discussed above. In this section we examine existing EU AI, liability and platform regulation frameworks to determine whether they provide a more feasible foundation to establish a legal duty to tell the truth for both narrow- and general-purpose LLM providers.

### 5.1. The EU's AI Act

The European Union's AI Act is Europe's first regulatory attempt to govern AI systems including generative AI. Unfortunately, the vast majority of the framework focuses on how to regulate predictive AI systems, such as high-risk systems^87^ deployed in criminal justice, employment or immigration. Chapters 2 and 3 establish duties such as technical documentation, record keeping and maintaining transparency, human oversight, accuracy, cybersecurity and robustness for providers of high-risk systems. According to Article 5 certain applications and use cases are deemed too risky to use and are banned, including emotion detection AI in schools or workplace, ‘social scoring’, and certain uses of real-time remote biometric identification. None of these rules apply directly to LLMs or generative AI unless used in a high-risk context.

Beyond these provisions, Article 50 establishes a transparency duty for deployers of certain AI systems including generative AI (e.g. Deepfakes, chatbots). Certain outputs have to be ‘watermarked’ and users must be informed that they are engaging with a chatbot. These requirements are to be welcomed, but critically they do not address the risks of careless speech. Being informed that an output is artificially generated does not provide any information about its factuality or truthfulness.

Articles 51 and 52 distinguish between general-purpose AI models, and general-purpose AI models with systemic risks. Article 53 and Annex XI establish a duty for providers of general-purpose AI models to draw up technical documentation (e.g. about training and testing results), establish certain transparency duties for the value chain, expect providers to respect copyright law and require reporting on the known or estimated energy consumption of models. These requirements for general-purpose models do not include a duty to design systems that generate truthful output.

Providers of general-purpose AI models with systemic risks—those with floating point operations (FLOPs) greater than 10^25^—face additional duties such as model evaluations and adversarial testing, mitigation strategies for systemic risks, reporting duties for serious incidents and corrective measures, and appropriate cyber security (Article 55). Again, none of these requirements equate to a public duty to speak the truth.

However, the technical standardization process being carried out by CEN/CENELEC for the AI Act will show how ‘systemic risks' are defined [140]. It is not unreasonable to assume that mis- and disinformation could be classified as a systemic risk of LLMs. However, this does not mean that careless speech will necessarily be seen as a systemic risk. Indeed, indications in Article 50 would suggest careless speech will not be classified as such, as it states that transparency about the outputs of generative AI (e.g. Deepfakes and chatbots) is sufficient to mitigate their risks [141].

### 5.2. Product liability directive and AI liability directive

The European Union is currently negotiating two new legal frameworks to complement the EU AI Act [142] that focus on liability rules around AI generated output: an update to the current Product Liability Directive (PLD) [143] and a new AI Liability Directive (AILD) [144]. These directives are intended to create an individual accountability mechanism for harmed parties. Despite this aim, it is unlikely that either directive will address and remedy the harms of careless speech.

The updated PLD widens the framework's existing scope to include harms caused by software and digital services (including AI), not just by products. Currently the directive only covers physical products and electricity. Despite this update the first draft of the revised framework was not equipped to manage careless speech harms because it is limited to providing compensation for material losses resulting from death, personal injury, damage to property or loss or corruption of data ([143] at Section 1.2). Immaterial and financial harms were not covered.^88^ The current updated draft now includes a compensation mechanism for certain ‘non-material’ harms which are currently covered by national law.^77^ However, this comes with a significant caveat: Recital 23 states that the PLD should provide ‘compensation for non-material losses resulting from damage covered by this Directive, such as pain and suffering…in so far as such losses can be compensated under national law’ [141]. In other words, ‘non-material’ harms such as careless speech only fall under the Directive if they are a side-effect of one of the listed material harms (e.g. destruction to property), and if the Member State laws also recognize the harms in question. Careless speech is not likely to occur because of death, personal injury, damage to property or loss or corruption of data of a product and even if so, it is questionable whether careless speech harms are currently recognized by Member State laws.

Similarly, the AILD is also unlikely to offer recourse mechanisms against providers and deployers of AI systems (Article 3(1)). The Directive only covers harms caused by fully automated AI systems, meaning minimal human involvement can render the Directive inapplicable ^89^ However, unlike the PLD, the AILD explicitly mentions fundamental rights violations in Article 2(9) and opens the scope for redress for immaterial harm [145].

Fundamental rights are not, however, definitely guaranteed. Their inclusion will depend on the interpretation of Member States in implementing the Directive (which can lead to a fragmented standard across the EU^90^). In the explanatory notes on the legal basis of the directive the European Commission lists several fundamental rights of the Charter as potentially falling within the scope of the directive. These include violations against personal dignity, respect for private and family life, right to equity and non-discrimination [144, p. 10]. As discussed above, truth duties and harms caused by careless speech do not fall under any of these fundamental rights (see §4.2).

Even if the Member States see careless speech as a harm worth preventing, the harms caused by the spread and production of careless speech do not fit cleanly into any existing fundamental rights. Much like environmental harms they are not immediate or individually tangible. The AILD requires a concrete material or immaterial harm resulting from a fundamental rights violation to qualify for redress, but careless speech harms will typically not meet these requirements. As discussed above, general-purpose LLM providers are currently under no obligation to build systems that tell the truth or produce accurate content (see §5) and so it is unlikely that people will have a redress mechanism against careless speech.

Assuming relevant fundamental rights and immaterial harms can eventually be covered by the new liability directives, they would still be unlikely to provide meaningful recourse for individuals and groups harmed by careless speech. The directives' individual recourse mechanisms are based solely on fundamental rights. As discussed above, fundamental and human rights law applies almost exclusively to public institutions except in rare cases with horizontal effects. This means that the new liability directives will not provide remedies against fundamental rights violations committed by private actors including LLM providers. This limitation of scope severely restricts the utility of these directives to mitigate harms for individuals and groups harmed by AI.

Assuming these barriers can be overcome through Member State interpretation, the process for individuals to bring a claim against public institutions and private actors is arduous. Claimants would need to prove (1) fault, (2) causality between the fault and an LLM output, and (3) causality between outputs and damage [141].

Evidence is key for proving a fundamental rights violation. The directives contain an important limitation in this regard: disclosure of evidence mechanisms only applies to high-risk AI systems as defined in the AI Act, but not other AI systems. A key distinction based on the scope of purpose of AI systems was added to the AI Act in the final stages of trilogue negotiations. Originally, the AI Act distinguished between AI systems based on their risk level: low risk, high risk and unacceptable risk. The latest version introduces a separate category for general-purpose models: (1) general purpose AI models and (2) general-purpose AI models with systemic risk.^91^ Critically, neither type of model is classified as high-risk by default, meaning the disclosure mechanisms of the AILD will not apply to general-purpose models, including LLMs, by default.^92^ Claimants will thus lack the ability to request evidence from LLM providers pursuant to fundamental rights violations.^93^

Alleviation of burden of proofs will be equally hard to trigger. The rebuttable presumption of causality between fault and output is only assumed if non-compliance with a duty laid down in the AI Act can be shown.^94^ As discussed above, LLM providers do not face duties to tell the truth and are thus not at fault for failing to live up to a relevant duty.

A rebuttable presumption of fault and therefore non-compliance with a relevant duty is for example granted if the defendant fails to disclose the evidence requested.^95^ This duty only exists for high-risk AI systems and it is unclear if LLMs will be classified as such by default. In any case, causality between the output of the system in question and damages still needs to be proven.

Even in the best possible circumstances it will be incredibly difficult for individuals to prove fault, causality and damage, even if technical documentation is supplied by LLM providers via disclosure mechanisms, and fault and causality are assumed. The main reason is that the harms of careless speech are difficult to measure, experience and quantify. Careless speech does not cause acute harms of the type regulated by the new liability directives. Inaccurate, non-representative or biased information does not cause immediate physical or psychological harm or cause financial losses, but rather leads to immaterial, cumulative damages over time.

### 5.3. Digital Services Act

The Digital Services Act, an update on the E-Commerce Directive, aims to regulate online content on intermediary service providers such as Internet platforms and search engines. Among other things the new framework aims to mitigate illegal speech (e.g. hate speech) and the spread of harmful speech such as disinformation and misinformation by clarifying liability rules for Internet platforms.

The DSA is not well equipped to deal with the harms of careless speech. It is unclear whether the DSA, which applies to all intermediary services, including Very Large Online Platforms (VLOPs) and Very Large Search Engines (VLSEs), will also apply to companies such as OpenAI that operate commercial LLM systems like ChatGPT. Second, the harms of careless speech do not neatly fit into the harms addressed by the framework and associated legal obligations. The DSA does not compel platforms and search engines to monitor and rectify incorrect outputs.

The DSA, like its predecessor the E-Commerce Directive, offers a liability privilege to intermediary services such as Internet platforms, search engines and other hosting services for the content they host. However, should these operators be made aware or become aware of illegal or otherwise harmful activity they are legally obligated to take action against it. The liability privilege hinges on the neutrality of the platform. To be free from liability platforms must only host or moderate content, but not actively create it.

As Hacker *et al*. [139, p. 1118] have convincingly argued, providers of LLMs (e.g. GPT-4) will not be considered service providers under the DSA because they are no ‘mere conduit’, ‘caching’ or ‘hosting’ services. While their exclusion means they cannot claim the liability privilege, it also means they are not bound by the same duties to prevent illegal speech or misinformation and disinformation. For example, VLOPs and VLSEs are required by the DSA to conduct external audits, create internal processes to mitigate and prevent misinformation, and should sign up to a voluntary code of conduct to curb the spread of misinformation [128, p. 5]. LLM providers will not be bound by these duties.

The DSA may nonetheless govern liability further along the LLM lifecycle. The usage of LLMs by social media platforms, search engines and other Internet platforms (LLM hosting platforms) would bring LLMs within the scope of the DSA.^96^ It may also render the liability privilege inapplicable to the platforms using them. These service providers would no longer solely be acting as a mere conduit, caching or hosting service but rather actively participating in its creation by hosting an LLM [139, p. 1118]. Of course their duties as service providers will remain (e.g. notice and takedown, external audits for VLOPs).

If this interpretation is correct VLOPs and VLSEs could be held directly liable for illegal speech, misinformation and disinformation produced by LLMs they host, rather than being treated as a neutral host of the content. Problematically, their abilities to modify, fine-tune or re-train LLMs will be limited in many cases when the platform itself is not the developer of the hosted LLM, meaning their ability to limit future illegal speech may be limited.

While the regulation of LLMs hosted by VLOPs and VLSEs via the DSA is a welcome step, the question remains as to whether the duties incurred are fit for purpose to mitigate careless speech harms.^97^ These harms do not neatly fit into the definitions of misinformation and disinformation found in DSA and associated codes of conduct. The EU European Democracy Action Plan (EDAP),^98^ which lays the groundwork for the Strengthened Code of Practice on Disinformation 2022 from which the DSA draws its definition of disinformation,^99^ defines misinformation and disinformation as follows:

Misinformation is false or misleading content shared without harmful intent though the effects can be still harmful, e.g. when people share false information with friends and family in good faith. Disinformation is false or misleading content that is spread with an intention to deceive or secure economic or political gain and which may cause public harm.^100^

The EDAP and related Code of Practice are predominantly concerned with misinformation and disinformation around the COVID-19 pandemic, elections and democratic processes (e.g. during the 2020 US election, Cambridge Analytica), radicalization of people and incitement of violence and propaganda and interference from foreign countries impacting Western democratic institutions.^101^ The DSA itself acknowledges other types of systemic harms in VLOPs and VLSEs such as illegal content,^102^ negative impacts on human rights, gender-based violence, violations of protections for public health and minors, and negative impact on physical and mental well-being (*Id.* at Article 34(1)(b-d)). Careless speech does not fit neatly into these definitions of misinformation and disinformation or the systemic harms described in the DSA.

Regardless of the applicability of the DSA, Internet platforms and companies such as OpenAI that provide users with direct access to LLMs are bound by speech regulations of the Member States. As discussed above (see §3.1), the harms of careless speech do not rise to the level of known codifications of speech regulations that govern acute physical, psychological or reputational harms (e.g. libel, slander). Careless speech harms also do not rise to the same level as criminal law such as hate speech because current EU law does not contain a general duty to speak the truth (see §4.2.1).

### 5.4. Google autocomplete case

While the AI Act, Product Liability Directive, AI Liability Directive and DSA are not well equipped to mitigate careless speech harms, the German Federal Court of Justice might offer some inspiration on how to deal with careless speech in the future. The ‘Google auto-complete case’ dealt with the question on whether Google's autocomplete function could infringe personality rights. In 2011, the chairman of a food supplement corporation filed a lawsuit against Google because autocomplete suggested ‘fraud’ and ‘Scientology’ when his name was entered. This was claimed to infringe the claimant's reputation because the search results suggested his connection with fraudulent activity and an association with the Church of Scientology, neither of which had any basis in fact. The claimant lost in the first^103^ and second instance,^104^ but the German Federal Court of justice eventually agreed that an infringement of his personality rights had occurred.^105^

This case is particularly relevant to problems of aligning LLMs and AI systems with truth. The Court was fully aware that the autocomplete function was not designed to make suggestions based in fact, but instead is influenced by a variety of factors such as prior search patterns of other users.^106^ Despite this, the Court decided that the autocomplete function is reputationally damaging because it suggested to users that the claimant is associated with ‘fraud’ and ‘Scientology’.^107^

A key argument made by the claimant was that Google does not provide randomly selected autocomplete suggestions. Rather, the purpose of the search engine is to help individuals find relevant results based on a search query. Users therefore have an expectation that autocomplete suggestions have some material connection with the search query.^108^ This expectation is not circumvented by the fact that autocomplete suggestions are based primarily on prior user queries and not ground truth. Further, Google is responsible for the display of the autocomplete suggestions because the company, not third parties, plays an active role in ranking and preparing suggestions for users.^109^

Two points are important to note. First, the Court's judgement unambiguously states that Google does not have an obligation to preventatively filter inaccurate information, but rather that they must only act once they have been made aware of inaccuracies.^110^ Actions must, however, be taken to prevent future similar infringements;^111^ this would be the equivalent of requiring LLM providers to fine-tune models to correct for known inaccuracies or hallucinations. This duty reflects the enforcement model of the DSA. A duty to pre-emptively guarantee accurate content, understood as a truth duty, cannot be derived directly from the judgement.

Second, the main reason the claim was successful was because autocomplete was seen as libellous and infringing the claimant's personality rights. Careless speech harms are less likely to rise to the same level as libel. It is therefore unclear whether the German Federal Court of Justice would also grant the same protection for outputs that are not reputationally damaging.

With these limitations in mind, this case remains highly relevant to truth duties for LLM providers because the claimant successfully argued that users have an expectation that a particular platform function, in this case autocomplete, faithfully reflects ground truth relevant to their search query. This holds even if people know that Google's search engine is not based on accurate information, but rather reflects the search behaviour of previous users.

The Court's ruling in this case provides a promising but narrow entry point for truth duties in LLMs. Recognizing that the Court believes that even an informed user can be tempted to believe that the outputs of autocomplete are accurate, it is reasonable to assume that the Court could grant similar protection against LLMs that produce careless speech. Autocomplete and LLMs share similar design elements: they are optimized to be convincing and helpful for users, are built or trained on a large corpus of Internet data (e.g. books, Wikipedia entries, forum posts), and outputs are fine-tuned or subjectively ranked based on user feedback and prior usage. Similarities extend to their intended uses as well. LLMs are being implemented in major search engines including Bing and Google to serve a similar function to autocomplete by suggesting answers to user queries and helping them refine their prompts to find better, more relevant information. Successfully extending the claimant's arguments and Court's interpretation in this case to create a truth duty for careless speech LLMs thus appears feasible.

## 6. A duty to minimize careless speech

Our analysis has shown that EU law contains few explicit regulations and duties to tell the truth. Where these duties exist they tend to be limited to specific sectors, professions or state institutions, and rarely apply to the private sector. The harms of careless speech are stubbornly difficult to regulate because they are intangible, long-term and cumulative.

Most of the reviewed regulatory frameworks were not designed with careless speech or technologies like LLMs in mind, and do not reflect their unique capacities to homogenize knowledge and automate work and speech that has traditionally required human intelligence. LLM providers are a type of actor that fall in a gap in existing legal frameworks. Current frameworks are designed to regulate specific types of platforms or people (e.g. professionals), but not to regulate a hybrid of the two.^112^ Future regulatory instruments need to explicitly target this middle ground between platforms and people. Liability regimes appear to provide the best and most stringent possible pathway forward to derive or create such a duty (see §6).

Despite this general regulatory landscape, the limited truth duties found in science and academia, education and libraries and archives offer an interesting avenue to explore as LLMs serve a similar function (see §4). Concerns about negatively impacting the economic freedom of LLM providers by establishing a duty to design truthful systems can be mitigated by acknowledging that the harms of careless speech cause individual and societal harm at large scale. Similar to the need to ensure sufficient protections for the environment, public health, internal and external public security, stability of financial market and cultural heritage, the prevention of careless speech harms is equally important due to its collective and societal harms. The public good of truthfulness, right to know the truth, and the duty to remember needs to be counterbalanced with competing economic interests of LLM providers (see §§3 and 5).

Providers of narrow- and general-purpose LLMs nonetheless bear responsibility for the growing spread of careless speech and its harms. In recognition of the significant risks posed by LLMs to shared social and scientific truth in society, we propose the creation of a legal duty to minimize careless speech for providers of both narrow- and general-purpose LLMs and derived commercial applications. The scope of this duty must be broad; a narrow duty would not capture the intangible, longitudinal harms of careless speech, and would not reflect the general-purpose language capacities of LLMs.

This duty emphasizes that no single entity, be it public or private, should be the sole arbiter of truth. The core risk of LLM-generated careless speech is that it transforms truth into a question of frequency and majority opinion, not fact or social reality. The immediate harm caused by careless speech is that it misinforms. Reflecting this and referring back to our procedural account of truth (see §3), the proposed duty to tell the truth is not intended to force alignment with a single authoritative body of knowledge or to priorities a positivist ontology, but rather to improve the epistemological rigour of LLMs (see §3) [88]. Our account of truth is procedural and focuses on epistemological requirements, for example how truth is investigated, debated and justified, without committing to a specific ontology.

It would require LLM providers to take steps to align their models and applications with ground truth and optimize for plurality and representativeness of sources. Truth-telling LLMs should be designed to produce outputs based on a diversity of source material, and not solely based on the frequency of statements in training data and opaque fine-tuning as is currently accepted practice.

The proposed duty requires transparent and accountable reporting to public institutions and civil society in how this alignment occurs, and engagement with local stakeholders on especially contentious topics to ensure a diversity of views are reflected in model outputs. Governance initiatives such as OpenAI's ‘Democratic inputs to AI’ grant programme, which funds teams to create stakeholder-led governance and fine-tuning models, are a step in the right direction.^113^ However, such initiatives must also be open to the public. The fine-tuning, model re-training and construction of ‘guardrails' carried out based on such stakeholder governance initiatives must involve the public and not be solely overseen by AI providers themselves to avoid centralized, private control of truth and acceptable speech in LLMs.

Tackling the difficult methodological challenges needed to make general-purpose LLMs reliably tell the truth is not an overriding requirement in current research and development of LLMs. The focus on building appropriate guardrails, eliminating toxic and sensitive content and preventing leakage of personal data through human feedback and fine-tuning is leading towards systems that tell, at best, a user-friendly, heavily moderated and relativistic version of the truth. It is the functional disregard for truth, or lack of a strict requirement or good faith intent to tell the truth however understood, which makes LLMs dangerous to science, education and society. This is not to suggest that truth is disregarded entirely in their development; the problem is instead that truthfulness is not an overriding design requirement or necessary precondition for ‘useful’ responses.

This is largely a problem of incentives. If models are built to maximize engagement and usability, empirical grounding and factual content are only of secondary importance. Current incentives to build guardrails focus not on making systems tell the truth, but rather reducing the liability of their developers and operators. Guardrails alone cannot change the path currently being taken, only make it safer. Current development pathways end with increasingly powerful, but stubbornly incidental, truth engines. The duty to minimize careless speech seeks to change paths and redirect development towards public governance of truth in LLMs.

## Data Availability

This article does not contain any additional data.

## Footnotes

1 For information on specific safety-related steps taken by Google, see [11].

2 Two examples of open letters signed by hundreds of generative AI companies, researchers, and ‘thought leaders’ [22]. For an overview of ideologies in this public discourse, see [23,24].

3 Evidence from psychology experiments suggests people are more likely to find fast and confident speech to be persuasive [25,26]. Other experimental evidence suggests RLHF annotators find ‘assertive’ sounding outputs more believable [7]; similarly, content that aligns with prior beliefs (referred to as ‘sycophancy’) is also preferred independent of factuality [8]. Further research is needed into the strength and generalizability of these initial links between the truth and user belief of LLM outputs. Assuming these findings are valid, it is a separate question as to whether such belief patterns will change as LLMs are more widely adopted and social views of them change over time.

4 It is worth noting, however, that RLHF and other data enrichment work are often highly opaque and secretive processes. Users will rarely have full awareness of how precisely models have been aligned with ground truth through human feedback. See ([4], p. 262, [47], p. 16, [5], pp. 6–8).

5 Creating such a corpus, assuming such a thing is possible, would require adopting an alternative, rigidly fixed conception of truth such as positivism. LLMs could then be trained or fine-tuned against it, identifying correct responses across the corpus through annotation or pattern recognition, perfectly matching responses to prompts, and removing randomness as a design feature [55]. Describes a rule-based AI system built on this premise.

6 See also experimental evidence from psychology which suggests people are more likely to find fast and confident speech to be persuasive [25,26].

7 For an interesting discussion of the pros and cons of using LLMs in science, see [58].

8 Speech harms are most frequently connected to laws concerning free speech. See for example [77,78].

9 By ‘factual prompt’, we mean any type of prompt that requires factual information to answer. The prompt may contain an explicit request for a factual answer (e.g. ‘What year was President John F. Kennedy assassinated?’ or implicitly require factual information as one component of a subjective response (e.g. ‘What is the best way to cook a steak?’ which implicitly asks for reference to real cooking techniques).

10 To count as careless speech generated text need not be explicitly presented as authoritative, objective or factually correct to users, but rather need only meet a ‘reasonable expectation’ threshold, meaning the content would reasonably be interpreted as truthful by an average user owing to its form and content. Outputs which make factual claims and lack linguistic signals of uncertainty, doubt, ignorance or explicit reference to accuracy or confidence intervals would normally meet this requirement.

11 See Mittelstadt *et al*. [30], p. 1830 provide an example of ChatGPT 3.5 incorrectly listing explicit consent as a necessary legal basis to process personal data under the General Data Protection Regulation.

12 Ret [72], p. 38 reflects on a personal experience with ChatGPT providing incorrect and misleading factual statements: ‘the chatbot incorrectly referenced Model Rule 2.3 because the language closest to the cited language that ‘A judge shall perform the duties of judicial office impartially, competently, and diligently’ is found in Rule 2.5(A), not Rule 2.3. Rule 2.5(A) actually states, ‘A judge shall perform judicial and administrative duties, competently and diligently.’ Additionally, the quoted language that the chatbot attributed to Comment [3] does not appear in the Comments to either Rule 2.3 or 2.5. Following that heads-up from Judge Reyes, I noted that the chatbot's reference to Model Rule 1.1 is misleading. The amendment making it clear that a lawyer has a duty to keep up with the changes in technology that impact the practice of law is in Comment [8] to Rule 1.1 of the ABA Model Rules of Professional Conduct, not the Model Code of Judicial Conduct (a minor detail, I guess)’. Interestingly, the author struggles with how to classify the subtle inaccuracies produced by the model which we argue can be treated as careless speech, saying ‘Still, I was unsure if I should classify these missed details as “making up facts” or “hallucinating an output”’.

13 Care is a fundamentally human concept which cannot and should not be attributed to LLMs directly. In claiming LLMs produce careless speech we do not intend to attribute agency, intent, or otherwise anthropomorphize LLMs. Rather, LLMs lack care in their outputs (or ‘speech’) due to their design. Careless speech results from decisions taken by LLM providers, not any type of artificial agency or intent. Tronto [81,82], for example, has examined care as a unique feature of human interpersonal relationships which gives rise to unique moral considerations and duties.

14 See [83] LLMs will comply with this type of request even though their training data contain only the raw text of webpages and not the URL that identifies each page. As such, any reference URL must be taken from another document that linked to the original material and cannot be directly related to the referenced content.

15 Each of the content-related deficiencies that define careless speech hide many complex epistemological and ontological considerations. We do not intend to prioritize any specific ontology, but rather to identify deficiencies that are broadly applicable to truth seeking activities. Careless speech is itself not an objective concept; observers can justifiably disagree on whether a particular LLM output is truly an example of careless speech. Our aim instead is to conceptualize the type of speech current LLMs produce as technological necessity due to their design, and which cannot be fully solved or eliminated through technical fixes if the underlying design of the systems as text-prediction engines trained on non-authoritative data remains unchanged.

16 See [33]; Similarly, McGoey [91] has examined the political value of ignorance, according to which claims about having the least knowledge about a phenomenon can have significant strategic value, for example to avoid liability.

17 For our purposes, we assume that LLMs are not moral agents and cannot be described as having intent or human-like agency. For a fuller exploration of this topic, see [92–94].

18 For more on this topic, see [104].

19 See Kidd & Birhane [16, p. 1222]. Knowledge and information are necessary for people to be independent, self-sufficient, critical, and to make well-informed decisions about their well-being. Careless speech makes individuals less well-informed and independent. People may be more likely to fall prey to other's bad intentions and or to understand when they are being manipulated as the quality of common truths degrade (see §3). These are not hypothetical concerns, as recently shown when a Dutch committed suicide to mitigate climate change following encouragement from an LLM-powered chatbot [106].

20 For an overview of the meaning of ‘accountability’ as applied to AI through legal and ethical duties, see [115].

21 See Everson & Gonçalves [123]. A quasi-subjective right refers to a right that occurs between two individuals.

22 Horizontal applicability will be the case when the provisions constitute a fundamental principle of the EU, such as the prohibition against discrimination based on nationality or unequal pay between men and women; see [123] at 266–267.

23 It should be noted that ECJ jurisprudence is often unclear and frequently silent on positive obligations under the Charter (e.g. under Article 11). For a discussion on ECJ jurisprudence and the literature on positive obligations, see [125].

24 See [125] at 349–50, 362, 366. According to Woods, it is doubtful that Article 11 has horizontal effects.

25 See [125] at 347. See for example on parody and copyright, Case C-201/13 Deckmyn, Vrijheidsfonds VZW v Vandersteen *et al*. (Judgment 3 September 2014).

26 See [126], https://brill.com/display/title/58950 (accessed 21 November 2023) and the case law (e.g. Couderc *et al.* v. France 2015) where the Court developed a test to assess whether information is libelous or in the public interest, including questions like: ‘How did the journalist obtain the information? Did the information prove to be true, and did the journalist act in good faith?’.

27 For case law on criminal law and publishing untrue statements, see [126] at 532.

28 Woods [125] at 348, 360 and see also Case C-345/17 Buivids v Datu valsts inspekcija (Judgment 14 February 2019) for an ECJ ruling on whether free speech or privacy protection should prevail.

29 See [125, pp. 359–360] describing that there can be exceptions if the topic is particularly divisive like the Holocaust or the Troubles in Northern Ireland.

30 For an overview of the case law of the ECJ and the ECHR, see [127].

31 Regulation (EU) 2015/2120 of the European Parliament and of the Council of 25 November 2015 laying down measures concerning open internet access and amending Directive 2002/22/EC on universal service and users’ rights relating to electronic communications networks and services and Regulation (EU) No 531/2012 on roaming on public mobile communications networks within the Union, 18 (2015), http://data.europa.eu/eli/reg/2015/2120/oj.

32 Must-carry rules are regulatory requirements which create obligations for cable television providers to broadcast certain locally licensed television stations. For must-carry rules in the EU, see Directive (EU) 2018/1972 of the European Parliament and of the Council of 11 December 2018 establishing the European Electronic Communications Code, 214 Article 114 (2018), http://data.europa.eu/eli/dir/2018/1972/oj.

33 Guerra and Others v. Italy—58135/09 (1998), https://hudoc.echr.coe.int/eng?i=001-58135 (accessed 31 January 2024).

34 See [126, p. 540] citing Schuman v. Poland (2014) para 24. The ECHR has interpreted Article 10 in a way that the enshrined rights to search and access information only apply to truthful information or to information that is at least communicated in good faith. See [126, p. 526] citing Salumäki v. Finland (2014) para 47 which calls for journalists to act in ‘good faith in order to provide accurate and reliable information’.

35 Villiger [126, p. 510] citing Lehideux *et al.* v. France (1998) para 46–58. A dispute between historians about the Pétain régime in World War II. See also Monnat v. Switzerland (2006) para 57 on Switzerland's role during World War II.

36 Peri̇nçek v. Switzerland—27510/08, para 209–212 (2013), https://hudoc.echr.coe.int/eng?i=001-139724 (accessed 31 January 2024).

37 Proceedings brought by Sergejs Buivids—Case C-345/17, (2019), https://eur-lex.europa.eu/legal-content/en/TXT/?uri=CELEX:62017CJ0345 (accessed 31 January 2024).

38 For example, the ‘Mediengesetz’ in Austria, see Medienprivilegien—Verband Österreichischer Zeitungen, https://voez.at/politik-recht/rechtsinformationen/medienprivilegien/ (accessed 31 January 2024).

39 Manole and Others v. Moldova—13936/02, para 100 (2009), https://hudoc.echr.coe.int/fre?i=001-94075 (accessed 31 January 2024).

40 Directive 2010/13/EU of the European Parliament and of the Council of 10 March 2010 on the coordination of certain provisions laid down by law, regulation or administrative action in Member States concerning the provision of audiovisual media services (Audiovisual Media Services Directive) (Codified version) (Text with EEA relevance), OJ L 95/1 (2010), http://data.europa.eu/eli/dir/2010/13/oj/eng (accessed 31 January 2024).

41 Directive 2000/31/EC of the European Parliament and of the Council of 8 June 2000 on certain legal aspects of information society services, in particular electronic commerce, in the Internal Market (‘Directive on electronic commerce’), OJ L 178/1 (2000), http://data.europa.eu/eli/dir/2000/31/oj/eng (accessed 31 January 2024).

42 Regulation (EU) 2022/2065 of the European Parliament and of the Council of 19 October 2022 on a single market for digital services and amending Directive 2000/31/EC (Digital Services Act) (Text with EEA relevance), 277 OJ L (2022), http://data.europa.eu/eli/reg/2022/2065/oj/eng (accessed 27 January 2024).

43 Case C-288/89 Gouda, Case C-368/95 Familiapress (for magazines) and Case C-283/11 Sky for ‘events of high interest to the public’ para 52. Stichting Collectieve Antennevoorziening Gouda and others v Commissariaat voor de Media - Case C-288/89, (1991), https://eur-lex.europa.eu/legal-content/EN/TXT/?uri=CELEX%3A61989CJ0288 (accessed 31 January 2024); Vereinigte Familiapress Zeitungsverlags- und vertriebs GmbH v Heinrich Bauer Verlag - Case C-368/95, (1997), https://eur-lex.europa.eu/legal-content/EN/TXT/?uri=CELEX%3A61995CJ0368 (accessed 31 January 2024); Sky Österreich GmbH v Österreichischer Rundfunk-Case C-283/11, para 52 (2013), https://eur-lex.europa.eu/legal-content/en/TXT/?uri=CELEX:62011CJ0283 (accessed 31 January 2024).

44 Woods [125, p. 368] Member State laws may, of course, have their own rules for truthful and pluralistic media content, but they are beyond the scope of our analysis.

45 Société Neptune Distribution v Ministre de l’Économie et des Finances - Case C-157/14, (2015), https://eur-lex.europa.eu/legal-content/en/ALL/?uri=CELEX%3A62014CJ0157 (accessed 31 January 2024).

46 Philip Morris Brands SARL and Others v Secretary of State for Health - Case C-547/14, (2016), https://eur-lex.europa.eu/legal-content/en/TXT/?uri=CELEX:62014CJ0547 (accessed 31 January 2024).

47 Policy concerning archival duties of the institutions of the European Union, see: Archival policy—European Commission, https://commission.europa.eu/about-european-commission/service-standards-and-principles/transparency/access-documents/information-and-document-management/archival-policy_en (accessed 31 January 2024). See also Council Recommendation of 14 November 2005 on priority actions to increase cooperation in the field of archives in Europe, 312 OJ L (2005), http://data.europa.eu/eli/reco/2005/835/oj/eng (accessed 31 January 2024) which aims to facilitate cooperation and coordination of the Member States for archival purposes.

48 For this and international and national law on archives, see [105].

49 In Austria, for example, the Bundesmuseen-Gesetz 2002 (Federal Museum Act 2002) regulates the Austrian National Library. The law contains provisions to maintain cultural heritage and foster scientific discourse and to keep accurate historical collections. See Rechtsgrundlagen, Sammelrichtlinien und Erklärung zu potenziell verletzenden Inhalten, | Österreichische Nationalbibliothek, https://www.onb.ac.at/mehr/ueber-uns/rechtsgrundlagen-sammelrichtlinien-und-erklaerung-zu-potenziell-verletzenden-inhalten (accessed 31 January 2024). Although not a Member State the UK has the Public Libraries and Museums Act 1964 which establishes in paragraph 7 the duty ‘of every library authority to provide a comprehensive and efficient library service for all persons desiring to make use thereof’ including keeping an accurate stock.

50 See [105], pp. 65, 74–6; Also reflected in [129].

51 For more on EU and ECHR actions to preserve cultural memory, see Andresen [105, p. 76].

52 European Commission v Hungary - Case C-66/18, para 227 (2020), https://eur-lex.europa.eu/legal-content/en/TXT/?uri=CELEX:62018CJ0066 (accessed Feb 1, 2024); see also [130].

53 See [130, p. 417]. The only exception to this rule is Art 2(b) of ‘Council Directive 2005/71/EC on a specific procedure for admitting third-country nationals for the purposes of scientific research’ which defines ‘research’. The Directive states that research can be undertaken by approved private as well as public entities (Article 2(c)) and defines research as ‘creative work undertaken on a systematic basis in order to increase the stock of knowledge, including knowledge of man, culture and society, and the use of this stock of knowledge to devise new applications’. While this is a helpful definition, the Directive only applies in relation to admitting nationals to work in the EU and thus the scope is limited.

54 European Commission v Hungary - Case C-66/18, [130].

55 See OECD [132] defines research as: ‘Research and experimental development (R&D) comprise creative and systematic work undertaken in order to increase the stock of knowledge—including knowledge of humankind, culture and society—and to devise new applications of available knowledge’. See [133].

56 European Commission Directorate-General for Research, [115] at Section 3.

57 European Commission v Hungary - Case C-66/18, [130] at para 225.

58 Frantziou & Mantouvalou [121, p. 460] and the cited case law. There is a duty to protect against undue influence from employers and trade unions.

59 Everson & Gonçalves [123, p. 477]. Michelle Everson and Rui Correia Gonçalves in Peer page 477.

60 It is worth noting that any obligations that impact Articles 15–17 must pass the proportionality test.

61 See [121, p. 459] and case law cited therein.

62 Consolidated versions of the Treaty on European Union and the Treaty on the Functioning of the European Union Protocols Annexes to the Treaty on the Functioning of the European Union Declarations annexed to the Final Act of the Intergovernmental Conference which adopted the Treaty of Lisbon, signed on 13 December 2007 Tables of equivalences, (2016), https://eur-lex.europa.eu/legal-content/EN/TXT/?uri=celex%3A12016ME%2FTXT (accessed 1 February 2024).

63 Mark Alemo-Herron and Others v Parkwood Leisure Ltd - Case C-426/11, para 33, 35 (2013), https://eur-lex.europa.eu/legal-content/en/TXT/?uri=CELEX:62011CJ0426 (accessed 1 February 2024) stating that employers cannot be forced to implement collective bargaining agreements in relation to pay lifts if they themselves have not been part of that negotiation. In the present case, the collective bargaining agreement was made before the jobs were transferred to Parkwood Leisure Ltd and an automatic transfer of prior agreements cannot be expected.

64 For this and a more detailed discussion on this issue, see [123, pp. 475–7].

65 International Transport Workers' Federation and Finnish Seamen's Union v Viking Line ABP and OÜ Viking Line Eesti - Case C-438/05, (2007), https://eur-lex.europa.eu/legal-content/en/TXT/?uri=CELEX:62005CJ0438 (accessed 1 February 2024) exploring the relationship of collective agreements and Article 16.

66 Laval un Partneri Ltd v Svenska Byggnadsarbetareförbundet, Svenska Byggnadsarbetareförbundets avdelning 1, Byggettan and Svenska Elektrikerförbundet - Case C-341/05, (2007), https://eur-lex.europa.eu/legal-content/en/TXT/?uri=CELEX:62005CJ0341 (accessed 1 February 2024) exploring the relationship between strike action and Article 16.

67 Scarlet Extended SA v Société belge des auteurs, compositeurs et éditeurs SCRL (SABAM) - Case C-70/10, (2011), https://eur-lex.europa.eu/legal-content/en/TXT/?uri=CELEX:62010CJ0070 (accessed 1 February 2024).

68 Belgische Vereniging van Auteurs, Componisten en Uitgevers CVBA (SABAM) v Netlog NV - Case C-360/10, (2012), https://eur-lex.europa.eu/legal-content/en/TXT/?uri=CELEX:62010CJ0360 (accessed 1 February 2024). In this case as well as in Scarlet extended the court explored possible legal obligations of ISP to implement monitoring software to prevent copyright infringement. Measures have to be balanced and not too costly for the ISPs. See also Tobias Mc Fadden v Sony Music Entertainment Germany GmbH - Case C-484/14, (2016), https://eur-lex.europa.eu/legal-content/en/TXT/?uri=CELEX:62014CJ0484 (accessed 1 February 2024).

69 Nycomed Danmark ApS v European Medicines Agency (EMA) - Case T-52/09, (2011), https://eur-lex.europa.eu/legal-content/en/TXT/?uri=CELEX:62009TJ0052 (accessed 1 February 2024).

70 Deutsches Weintor eG v Land Rheinland-Pfalz - Case C–544/10, (2012), https://eur-lex.europa.eu/legal-content/en/TXT/?uri=CELEX:62010CJ0544 (accessed 1 February 2024).

71 Wollenschläger [120, p. 513] and the cited cases therein. The ECJ has previously ruled against measures in areas such as transport, agriculture, foreign and security policy, public spending and environmental and economic policy.

72 Wollenschläger [120] and the cited case law therein. The full list of justifications as developed through ECJ jurisprudence is as follows: seizing funds to combat terrorism, measures for peace and international security, measures to secure the stability of the banking system, access to environmental information about companies, limiting the use of property in order to protect the environment, rules on emissions, freezing assets to prevent crime in the European Union, public security and insolvency, common and foreign security policy, measures in relation to agricultural policy, protection of cultural heritage, protecting health, consumer protection, maintaining peace and international security, protecting democracy, the rule of law and human rights, fostering the development of developing countries including the aim of eradicating poverty, measures against public spending in the context of financial and economic crisis, environmental protection, and any freedoms derived from fundamental freedoms or international obligations of the European Union.

73 Villiger [126, p. 540] citing Editorial Board of Pravoye Delo *et al.* v. Ukraine (2011) para 63.

74 Tietosuojavaltuutettu v Satakunnan Markkinapörssi Oy and Satamedia Oy - Case C-73/07, para 61 (2008), https://eur-lex.europa.eu/legal-content/en/TXT/?uri=CELEX:62007CJ0073 (accessed 1 February 2024); Proceedings brought by Sergejs Buivids - Case C-345/17, footnote 37 at para 53 as cited in; Woods [125] 140 at 352. For further discussion, see Are bloggers and YouTubers journalists?, European Intellectual Property Review (2020), https://www.westlaw.com/Document/I114E2FD02A0211EB9EEEC5D73ED9FAC8/View/FullText.html?transitionType=Default&contextData=(sc.Default)&VR=3.0&RS=cblt1.0&sp=wlnintell-000 (accessed 1 February 2024).

75 Proceedings brought by Sergejs Buivids - Case C-345/17, footnote 37 at para 55.

76 Proceedings brought by Sergejs Buivids - Case C-345/17, footnote 37 at para 70(2).

77 Rebechenko v. Russia - 10257/17, (2019), https://hudoc.echr.coe.int/eng?i=001-192468 (accessed 1 February 2024) See para 30 explaining ‘he was ordered to delete the video, publish a retraction, and pay about 714 euros (EUR) in non-pecuniary damage. The Court notes that these sanctions could discourage the participation of the press in debates on matters of legitimate public concern’.

78 Article 1(c) of the AVMSD defines editorial responsibility as ‘the exercise of effective control both over the selection of the programmes and over their organization either in a chronological schedule, in the case of television broadcasts, or in a catalogue, in the case of on-demand audiovisual media services. Editorial responsibility does not necessarily imply any legal liability under national law for the content or the services provided’.

79 Article 1(b) of the AVMSD defines a programme as ‘a set of moving images with or without sound constituting an individual item within a schedule or a catalogue established by a media service provider and the form and content of which are comparable to the form and content of television broadcasting. Examples of programmes include feature-length films, sports events, situation comedies, documentaries, children's programmes and original drama’.

80 It could nonetheless be informative to explore how advertising regulations and codes of ethics can or should be applied to generative systems given their shared ability and goal to influence people.

81 On the issues of keeping records trustworthy in practice, see Andresen [105] 117 at 84–6.

82 UNESCO [131] at Article 1(e). Specifically, the recommendation addresses institutions of higher education which ‘means universities, other educational establishments, centres and structures of higher education, and centres of research and culture associated with any of the above, public or private, that are approved as such either through recognized accreditation systems or by the competent state authorities’; European Commission v Hungary - Case C-66/18, footnote 52 also cites; Parliamentary Assembly of the Council of Europe, *Recommendation 1762 (2006). Academic Freedom and University Autonomy*, (2006), https://assembly.coe.int/nw/xml/XRef/Xref-XML2HTML-en.asp?fileid=17469&lang=en (accessed 1 February 2024) which speaks of autonomy of universities and rights and duties of researchers but only applies to universities.

83 European Commission Directorate-General for Research, OECD [132, p. 28] defines its scope as follows: ‘More specifically, this Recommendation relates to all persons professionally engaged in R&D at any career stage 18, regardless of their classification. This includes any activities related to ‘basic research’, ‘strategic research’, ‘applied research’, experimental development and ‘transfer of knowledge’ including innovation and advisory, supervisory and teaching capacities, the management of knowledge and intellectual property rights, the exploitation of research results or scientific journalism’.

84 European Commission v Hungary - Case C-66/18, footnote 52.

85 Gori [137, p. 437] citing Folger ø and others v Norway [GC] App no 15472/02, ECHR 2007-VIII.

86 As interpreted by the ECJ, public interests centre on public health, economic and financial stability, workers’ rights, environmental protection, internal and external public security, agricultural policy, protection of cultural heritage, consumer protection, and protecting the democracy and human rights. Consumer protection might be the closest that would allow restrictions, but it is unclear whether consumer protection law currently covers careless speech harms.

87 These are listed in Annex III of the AI Act.

88 For an extensive and excellent overview of further limitations of this Directive, see [145].

89 On how a token ‘human in the loop’ can render laws inapplicable, see [146].

90 For differing interpretations of data protection laws, see [146, p. 96].

91 This observation glosses over the important distinction between AI ‘models’ and ‘systems’ in the AI Act. Clear definitions are not provided in Article 2, but throughout the Act speaks of ‘AI systems’, ‘general purpose AI models, and ‘general purpose AI systems’. Confusingly, many definitions are requirements speak of providers of AI systems or general-purpose AI models, but fail to mention providers of general purpose AI systems. The significance of the distinction at this stage of implementation remains largely unclear, but may be intended to signify the difference between a general purpose model used for research and development, and a general purpose system intended for usage by end-users. For our purposes, we assume that rules for both general purpose AI models and general-purpose AI systems can apply to general-purpose LLMs in principle, and the classification of an LLM as a model or system will depend on the specific model of deployment or use case.

92 It should be noted that this gap could be remedied in future drafts of the AI Liability Directive which is currently going through trilogue negotiations. The category of ‘general purpose AI models’ and ‘general purpose AI models with systemic risk’ did not exist in the AI Act when the AI Liability Directive was originally proposed, so its absence at this stage of negotiations is expected.

93 These changes in cases where a general purpose model is used in a high-risk sector such as criminal justice or employment if there is also clear significant risk of harm to the health, safety or fundamental rights of natural persons. However, the harms we envision do not happen in the high-risk settings listed in Annex III of the AI Act. For more on how this shrinks the scope of Annex III, see Wachter [141].

94 European Commission [142] at 13 Articles 4(2-3) for high-risk systems or Article 4(5) for non-high-risk systems in the AI Act. These alleviations would only trigger if it would be ‘excessively difficult’ for the claimant to prove causality. This will be the case for example when an opaque system is used. See page 13 of the draft AI Act.

95 European Commission [142]. On the rebuttal presumption, see Article 3(5). Relevant duties that can be violated are outlined in Article 4(2-3). See also Hacker [145, p. 18].

96 For example, Meta has recently announced plans to implement LLM-based AI chatbots on Facebook, WhatsApp, and Instagram. Naomi Nix & Will Oremus, *Meta's AI Chatbot Is Coming to Social Media. Misinformation May Come with It*, Washington Post, 19 April 2024, https://www.washingtonpost.com/technology/2024/04/18/meta-ai-facebook-instagram-misinformation/ (accessed 9 May 2024).

97 A further gap in coverage of the DSA should be noted. Creating and sharing misinformation and disinformation is not illegal in itself. The DSA lacks obligations for platform users. Users flagged as contributing to misinformation and disinformation may face termination of their accounts or moderation of their posted content, but will not have broken the law. Even if such curbs on user behaviour existed, the envisioned victims and perpetrators of careless speech are not the same as foreseen by the DSA. Careless speech consists of subtly incorrect, non-representative or biased information. Unlike disinformation, it is not designed to deceive users or cause public harm. Careless speech need not be outrageous, scandalous, controversial or even blatantly wrong. Users will frequently be unaware that they have encountered careless speech because the mistakes at hand are subtle or require specific expertise to detect (see: §3).

98 Communication from the Commission to the European Parliament, the Council, the European Economic and Social Committee and the Committee of the Regions on the European democracy action plan, (2020), https://eur-lex.europa.eu/legal-content/EN/ALL/?uri=CELEX%3A52020DC0790 (accessed 27 October 2023).

99 2022 Strengthened Code of Practice on Disinformation | Shaping Europe's digital future, (2022), https://digital-strategy.ec.europa.eu/en/library/2022-strengthened-code-practice-disinformation (accessed 26 January 2024).

100 Communication from the Commission to the European Parliament, the Council, the European Economic and Social Committee and the Committee of the Regions on the European democracy action plan, footnote 97 at 18.

101 *Id.* at 18 defines foreign interference as ‘coercive and deceptive efforts to disrupt the free formation and expression of individuals' political will by a foreign state actor or its agents’. Similarly, ‘information influence operation refers to coordinated efforts by either domestic or foreign actors to influence a target audience using a range of deceptive means, including suppressing independent information sources in combination with disinformation’.

102 Regulation (EU) 2022/2065 of the European Parliament and of the Council of 19 October 2022 on a Single Market For Digital Services and amending Directive 2000/31/EC (Digital Services Act) (Text with EEA relevance), footnote 42 at Article 34(1)(a).

103 LG Cologne, 19 October 2011 - 28 O 116/11, (2011).

104 OLG Cologne, 10 May 2012 - I-15 U 199/11, (2012).

105 BGH, 14 May 2013 - VI ZR 269/12, (2013).

106 *Id.* at Paragraph 1 ‘Die im Rahmen dieser Suchergänzungsfunktion angezeigten Suchvorschläge werden auf der Basis eines Algorithmus ermittelt, der u.a. die Anzahl der von anderen Nutzern eingegebenen Suchanfragen einbezieht’.

107 *Id.* at Paragraph 15 ‘Verbindung ist geeignet, eine aus sich heraus aussagekräftige Vorstellung her vorzurufen’.

108 *Id.* at Paragraph 20 ‘Aus dem ‘Ozean von Daten’ werden dem suchenden Internetnutzer von der Suchmaschine der Beklagten nicht x-beliebige ergänzende Suchvorschläge präsentiert, die nur zufällig ‘Treffer’ liefern. Die Suchmaschine ist, um für Internetnutzer möglichst attraktiv zu sein - und damit den gewerblichen Kunden der Beklagten ein möglichst großes Publikum zu eröffnen - auf inhaltlich weiterführende ergänzende Suchvorschläge angelegt. Das algorithmusgesteuerte Such programm bezieht die schon gestellten Suchanfragen ein und präsentiert dem Internetnutzer als Ergänzungsvorschläge die Wortkombinationen, die zu dem fraglichen Suchbegriff am häufigsten eingegeben worden waren. Das geschieht in der - in der Praxis oft bestätigten - Erwartung, dass die mit dem Suchbegriff bereits verwandten Wortkombinationen - je häufiger desto eher - dem aktuell suchenden Internetnutzer hilfreich sein können, weil die zum Suchbegriff ergänzend angezeigten Wortkombinationen inhaltliche Bezüge widerspiegeln’.

109 *Id.* at Paragraph 22 ‘Sie hat mit dem von ihr geschaffenen Computerprogramm das Nutzerverhalten ausgewertet und den Benutzern der Suchmaschine die entsprechenden Vorschläge unterbreitet. Die Verknüpfungen der Begriffe werden von der Suchmaschine der Beklagten und nicht von einem Dritten hergestellt. Sie werden von der Beklagten im Netz zum Abrufbereitge halten und stammen deshalb unmittelbar von ihr’.

110 *Id.* at Paragraph 36 ‘Eine entsprechende präventive Filterfunktion kann zwar für bestimmte Bereiche, wie etwa Kinderpornographie, erforderlich und realisierbar sein, sie vermag jedoch nicht allen denkbaren Fällen einer Persönlichkeitsrechtsverletzung vorzubeugen’. ‘Weist ein Betroffener den Betreiber einer Internet-Suchmaschine auf eine rechtswidrige Verletzung seines Persönlichkeitsrechts hin, ist der Betreiber der Suchmaschine verpflichtet, zukünftig derartige Verletzungen zu verhindern’.

111 *Id.* at (c) ‘Weist ein Betroffener den Betreiber auf eine rechtswidrige Verletzung seines Persönlichkeitsrechts hin, ist der Betreiber verpflichtet, zukünftig derartige Verletzungen zu verhindern’.

112 For example, a medical website such as WebMD containing passive information derived from medical textbooks, scientific articles, and other sources which also allows users to ask questions and receive responses, is currently regulated to some extent. Individuals filling comparable roles (e.g. medical professionals) are also well regulated. A medical LLM designed to be used by clinicians or patients falls between these two categories. They give advice to users based on a variety of passive sources (akin to WebMD) in a more direct, convincing and personalized way comparable to medical professionals.

113 Democratic inputs to AI grant programme: lessons learned and implementation plans, https://openai.com/blog/democratic-inputs-to-ai-grant-program-update (accessed 25 January 2024).

114 See https://chat.openai.com/ (accessed 11 August 2023).

115 See https://chat.openai.com/ (accessed 11 August 2023). As of 9 May 2024 the disclaimer has been updated to: ‘ChatGPT can make mistakes. Consider checking important information’.

116 Text quoted from: https://bard.google.com (accessed 11 August 2023).

117 Text quoted from: https://bard.google.com (accessed 11 August 2023).

118 Text quoted from: https://gemini.google.com/app (accessed 9 May 2024).

119 Text quoted from: https://gemini.google.com/faq (accessed 9 May 2024). Interestingly, Gemini's FAQ discourages users from viewing the application as human or sentient, and using it to make important decisions: ‘Gemini isn't human. It doesn't have its own thoughts or feelings, even though it might sound like a human. Remember: Gemini can't replace important people in your life, like family, friends, teachers, or doctors. Gemini can't do your work for you. Gemini can't make important life decisions for you’.

120 Text quoted from: https://www.bing.com/new?scdexwlcs=1&showwlerror=1 (accessed 11 August 2023).

121 Text quoted from: https://www.microsoft.com/en-us/bing?form=MA13FJ (accessed 11 August 2023). Bing has since been renamed to Copilot. FAQ content relating to factual queries remains unchanged.

122 Text quoted from: https://ai.meta.com/llama/ (accessed 11 August 2023). Meta has since released LLaMa 3.

123 Text quoted from: https://ai.meta.com/llama/use-policy/ (accessed 11 August) 2023.
